# A Global Perspective: Reframing the History of Health, Medicine, and Disease

**DOI:** 10.1353/bhm.2015.0116

**Published:** 2015

**Authors:** Mark Harrison

**Keywords:** globalization, imperialism, modernity, global health

## Abstract

The emergence of global history has been one of the more notable features of academic history over the past three decades. Although historians of disease were among the pioneers of one of its earlier incarnations—world history—the recent “global turn” has made relatively little impact on histories of health, disease, and medicine. Most continue to be framed by familiar entities such as the colony or nation-state or are confined to particular medical “traditions.” This article aims to show what can be gained from taking a broader perspective. Its purpose is not to replace other ways of seeing or to write a new “grand narrative” but to show how transnational and transimperial approaches are vital to understanding some of the key issues with which historians of health, disease, and medicine are concerned. Moving on from an analysis of earlier periods of integration, the article offers some reflections on our own era of globalization and on the emerging field of global health.

For the past two decades, “globalization” has excited great controversy. There are conflicting opinions as to what it is, when it started, and its implications for humanity.^1^ Historians came relatively late to these debates but their contributions have been substantial, having helped to define and explain globalization, while providing instructive analogies with the past.^2^ Globalization has also had a significant impact on historiography. It has[Other P-639] spawned an entirely new field of enquiry—global history—but while its initial purpose was to comprehend the present, the remit of global history has expanded. Many historians have found a global perspective to be liberating, allowing them to pursue their interests beyond conventional boundaries of time and space.^3^ And yet, the “global turn” has its critics. Some claim that history written on such a scale lacks nuance, specificity, and rigor. Others fear that current preoccupations (globalization) will distort our treatment of the past. Differences and divergences may also be neglected in our eagerness to trace connections and shared experiences.^4^ Furthermore, it is argued, some global histories have tacitly endorsed the more exploitative aspects of globalization—an accusation recently leveled at some historians of medicine.^5^

But surprisingly few works in the history of health, disease, and medicine can accurately be described as global histories or claim to be such. Most are framed by geopolitical entities such as the nation-state or the constructs we term medical traditions. There is nothing wrong with writing history in this way, but if that is all we do we will miss an opportunity to speak to some of the most pressing issues of our time. A global perspective may also enable us to produce the interpretative syntheses that are such a rarity in our field.^6^ This article offers an account of the modern history of health, disease, and medicine that draws inspiration from the field of global history. Its principal aim is to show the relationship of these subjects to global dynamics, chiefly the emergence and development of modern world economies.

## Globalization and Global History

While global history began as a response to globalization, it is no longer defined by it. Most of its practitioners distinguish between their enterprise and the study of globalization, even if the latter is sometimes an object of their research.^7^ Moreover, few insist on globality in a strict, geographical sense. They conceive of “the global” in methodological and theoretical[Other P-640] terms, placing their subjects in frameworks that transcend conventional boundaries.^8^ The essential characteristic of global history—if it has one—is that it traces the networks that connect people and places, allowing historians to dispense with rigid compartmentalization. In these respects, global history has much in common with “transnational” or “connected” histories, though the former is obviously limited to periods and places in which nation-states exist.^9^ Global history also offers historians a standpoint from which long-term historical processes can be more easily discerned. Historians of health, disease, and medicine have not taken this opportunity as often as they might, and the result has been a historiography that is fragmented and with strong interpretative biases. The analysis of relatively small temporal or geographical units inclines toward histories of singularity and change, whereas more expansive frameworks tend to reveal connections and continuities. Both are clearly necessary, and it is time that the balance is restored.

In its early incarnations, global history concentrated chiefly on globalization and for this reason it was sometimes distinguished from world history, which emphasized the peculiarities of states and civilizations, as well as their internal dynamics of change.^10^ But this distinction is no longer valid, if it ever was. World histories *tend* to have a greater geographical and chronological scope than global histories. They are also more inclined to compartmentalize societies and cultures, many studies having been concerned with the “rise” (and demise) of “the West,” for example.^11^ Yet, most world historians have not allowed their frameworks to become static, and some are just as concerned with connectivity and integration as those who consider themselves globalists.^12^ It therefore makes little sense[Other P-641] to insist that global history and world history are distinct modes of analysis,^13^ especially now that the scope of global history has widened. Many works incorporate elements of both—focusing on points of interaction between global and “local” dynamics, marrying histories of connectivity with histories of structural change. This is very much the approach that I advocate. In what follows I argue that the rise and transmutation of the global market had a profound effect upon health, disease, and medicine, including the ways in which these concepts were understood and evaluated. While the results of these changes have already been considered in studies of individual colonies and nations, we have not sufficiently appreciated the relationship between them and forces that are global in nature.

This article examines global integration over a fairly long time scale, but it is not an attempt to trace the origins of globalization. Some historians do regard such an enterprise as legitimate and claim to find the antecedents of contemporary globalization deep in the past.^14^ But the quest for “archaic” or even “ancient” globalization makes me uneasy because it often entails inaccurate parallels with earlier periods. For this reason, I use the term “globalization” to refer only to the decades after 1970. Nevertheless, I recognize that earlier periods do bare some resemblance to globalization, in that one can observe connections that had acquired a degree of stability and affected large numbers of people.^15^ It is difficult to summarize these developments, but the process of integration considered in this article originated in the fifteenth and sixteenth centuries, in the “expansion” of Europe and the exploitation of a chiefly Afro-American “periphery” by a European “core.”

The initial stimulus for this expansion and the subsequent creation of a European “world system” was the quest for additional wealth and manpower following plague and environmental crises in Europe.^16^ However, while imperialism was the chief driver of integration, it was not the only one. European expansion was accompanied or preceded by that of other[Other P-642] peoples including the Arabs and Chinese.^17^ In many parts of Asia, Europeans capitalized their activities with the assistance of such communities, as well as with indigenous merchants and bankers.^18^ Similarly, they had to compete with commercial monopolies such as that established by the Ottomans in the Indian Ocean. Forces of integration were also at work beyond the empires of Europe. Although some of the old empires of Asia experienced stagnation and decay, others, like that of the Safavids, were forged anew. Indeed, the majority of people remained subjects of polities that were only tenuously linked to European systems. Confined to small trading concessions on the coasts and major rivers, Europeans had relatively little power or influence.^19^ The same was true, of course, of Japan, for it was not until the mid-nineteenth century that it was fully opened to the West.

But European imperialism was a radically disruptive force. During the next hundred years—through to roughly the middle of the nineteenth century—some of the great early modern empires, such as that of the Mughals, were fatally weakened, a process fomented and exploited by Europeans.^20^ Simultaneously, European countries such as Britain and the Netherlands enhanced their productive capacity in agriculture and industry, developed new financial institutions such as central banks and stock markets, and experienced a rise in consumer demand that subsequently fueled commercial expansion.^21^ European conflicts—like the Seven Years’ War (1756–63)—assumed global proportions and provided a further stimulus to empire building. By the nineteenth century, new technologies of communication such as steam propulsion and the electric telegraph enabled disparate colonial possessions to be welded into relatively cohesive empires.^22^ Around these, a global economy began to emerge, embracing parts of the world not formally under imperial control.[Other P-643]

The global dimensions of this new economy are indicated not only by the volume and variety of commercial transactions but by long-distance labor migration and the harmonization of prices for certain commodities.^23^ In recognition of their growing interdependence, countries sought agreement on the management of transportation and communications, aiming to reduce the impediments presented by national boundaries.^24^ However, the dynamics of global integration were complex, and the growth of international laws and institutions masked increasing competition and rivalry. This was particularly evident in agriculture, where improvements in transportation and new technologies such as refrigeration enabled New World farmers to undercut producers in Europe. Cheap meat and processed foods flowed into European markets, spurring calls for protection. In Europe itself, the rapid rise of Germany intensified imperial rivalry and strengthened those forces that urged self-reliance and trading preferences for their own empires.^25^

These rivalries provided the conditions for a global war in 1914–18, stalling the process of integration and reconfiguring international politics. After the war, a new spirit of internationalism, epitomized by the League of Nations, briefly flourished but soon foundered on political and economic realities.^26^ The rise of the Soviet Union, following the revolution of 1917, altered the political landscape and added an ideological dimension to international affairs. The onset of the Great Depression sharpened these divisions, while creating demands for economic protection and self-sufficiency.^27^ The rise of Nazi Germany and Japan’s pursuit of hegemony in Asia created the conditions for another major conflict, disrupting or severing many of the ties that had formerly existed. The war marked the end of fascist imperial ambitions and hastened the decline of the empires assembled by Britain and France.[Other P-644]

As this period of conflict came to an end, a new phase of integration began. After 1945, a concerted attempt was made to construct a new world order, the most prominent symbol of which was the United Nations. But integration proceeded for the most part within ideological blocs, capitalist and communist, and within them, too, significant divisions emerged. However, other important changes, including decolonization, the rise of multinational corporations, foreign aid, and the emergence of new forms of transnational consumerism, laid the foundations of contemporary globalization. This new era began in the 1970s, when the oil crisis hastened the collapse of “Fordist” methods of production and heavy industries in the West. The Soviet Bloc later began to exhaust its resources in competition with resurgent and ideologically driven Western nations, while the process of dialogue initiated by President Nixon in the early 1970s saw the gradual opening of China and the beginning of market reforms. The fall of the Soviet Bloc in 1989–91 permitted economic integration to proceed apace, harnessing new communication technologies, most obviously the Internet. The revolution in IT also had the effect of altering perceptions of time and space, both of which were compressed.^28^ Capital became extraordinarily mobile, and production was organized on a global basis. In tandem with these developments, a new economic doctrine—“neoliberalism”—began to dominate, portraying the state and organized labor as drags on efficiency. The effect was at once liberating and disorientating. Social and cultural forms that had existed for generations were rapidly dissolved, to be replaced by more cosmopolitan habits and trends. This complex, unstable world, marked by cruelly ironic juxtapositions of poverty and wealth, is the one we inhabit today.^29^

In the remainder of this article I show how these successive periods of integration have shaped not only the global contours of disease but the rise and expansion of Western medicine and concepts of health. By the twentieth century, some traditional medicines and medical products also came to be distributed on a global basis. But Western medicine was the chief beneficiary of global integration and arguably still is, even in countries known for their distinctive medical traditions. These developments are considered in four sections. The first three examine disease, medicine, and health up to the late twentieth century, while the last charts the effects of globalization since the 1970s. I have chosen to consider the[Other P-645] recent past separately because the sources on which my analysis is based are different from those of previous sections. While the first three are able to draw on an abundance of historical scholarship, coverage of the recent past is sketchy, and most of the works cited in the final section are utilized as primary sources to make provisional observations on the consequences of globalization.

## Disease

Pathogens know no borders and lend themselves to histories that are global or transregional in scope, Alfred W. Crosby’s *Columbian Exchange* and William H. McNeill’s *Plagues and Peoples* being some of the best known examples.^30^ These monographs made a deservedly large impact, not simply on account of their range but because their subjects were woven into the larger tapestry of history. Their thesis was that disease (and other biological agents in Crosby’s case) had shaped the destiny of civilizations, playing a crucial part in such momentous changes as the demise of feudalism and the Iberian conquest of South and Central America. Since then, the field of disease history has become crowded, to say the least. These works are too numerous to mention, and to review them would detract from my argument.^31^ However, we need to think more deeply about the ways in which historians of disease have envisaged long-distance connections, what their limitations are and how they might be addressed. In this section I argue that disease histories that fail to take into account structural and ecological change gravely oversimplify the relationship between disease and global integration. As George Rosen once put it, “Nowhere does human disease occur as ‘pure nature’; instead it is ever mediated and modified by social activity and the cultural environment which such activity creates.”^32^ We therefore need to find methodologies that help us to understand the complexities of disease transmission and to explain the long-term effects of integration on human and animal health. This means[Other P-646] examining not just the movement of pathogens but the transformation of social and ecological conditions—changes that affected indigenous as well as recently imported infections. Nor should we neglect the affects of global integration upon lifestyles and the diseases arising from changing patterns of work or cultural preferences. These are all important facets of “globalization,” today and in centuries past.

The deficiencies of a simple microbe importation model have long been recognized by students of European imperialism, and Crosby and McNeill have often been criticized for their “biological determinism.”^33^ Some have even suggested that they exaggerated the role of disease in historical change, particularly in the collapse of Native American civilizations.^34^ Although there is plenty of evidence to support the basic propositions of Crosby and McNeill,^35^ the charge of determinism has stuck, and many historians have subsequently stressed the social and political determinants of mortality. For example, the long-term impact of disease on the South Pacific islands has been shown to depend crucially on the nature of imperial rule. Where settler capitalism dominated—in other words, where indigenous peoples were dispossessed of their lands—populations took far longer to recover than in those colonies in which Europeans formed a small managerial elite.^36^ But the debate is not quite as polarized as this brief summary suggests. The “determinism” of Crosby and McNeill is often tempered by an acknowledgment that cultural and political factors played some part in population decline.^37^ Similarly, most social historians accept that disease has been an important and sometimes transformational historical agent.^38^[Other P-647]

The real question is where the emphasis should lie: how much weight should be given to pathogens and vectors, and how much to the forces that condition them? The answer depends on the case to be examined, but, in general, we can learn much from the approach taken by John R. McNeill in his recent study of the Caribbean.^39^ McNeill argues that the geopolitical history of the region was shaped by its disease ecology but that this environment was itself produced by human action. He shows how pathogens and disease vectors were shipped from Africa to the Americas on slaving vessels and how they became naturalized in environments profoundly altered by the rise of plantation agriculture. Although disease figures prominently in McNeill’s account as a force for historical change (and stasis), he stresses the coevolution of the slave-based economies and the biological agents that came to be embedded in them.^40^

McNeill’s work indicates how we might go about writing a global history of disease, a history that gives due attention to biological and political factors. But it would be impossible to undertake a similarly fine-grained analysis for a larger area, and so far there has been little appetite for similar research on other regions. Regional studies of disease in Europe are largely confined to medieval visitations of the plague, later known as the Black Death.^41^ One reason for this, perhaps, is the reluctance of many historians to indulge in speculation about the identity of diseases in the past—something that is essential to the political-ecological approach favored by McNeill. While there has been a protracted debate about the identity of European plagues in the medieval and early modern periods, this issue has also arisen in respect of the “Great Pox.” Some historians regard the use of the term “syphilis” to describe this malady not only as anachronistic but as illegitimate because it implies the existence of a stable entity over many centuries.^42^

If our aim is solely to understand disease as contemporaries did, then its biological identity is relatively unimportant. But the insistence that we must avoid using modern disease categories prevents us from charting the spread of disease or explaining the rise and fall of epidemics and their[Other P-648] relationship to economic, political, and environmental changes. These are surely legitimate questions. They are also vital if we are to attempt anything more than a localized study. Otherwise, there would be no reason to place the “French disease” or the “Neapolitan disease” within the same analytical frame, let alone *firangi roga* or any of the other names used to describe syphilis in Asia. If cross-cultural comparisons are to be attempted, or long-distance connections explored, then it is clearly useful to establish the identity of the disease in question. It is not always possible to do so with certainty, but analysis of ancient and modern DNA and stable isotopes is improving rapidly, and there are now many techniques that enable us to determine the existence of pathogens in the past.^43^ Such methods have already proved the existence of plague (as we would understand it) in medieval and early modern Europe and may yet settle other controversial issues such as the origins of the “Black Death,” which have been variously traced to China and Central Asia.^44^

Bio-archaeological and paleogenetic techniques will assuredly become important tools for those who wish to write the history of disease from a global or long-term perspective, and will be particularly important where manuscript and other documentary sources are fragmentary or ambiguous. This may be one reason why the disease history of parts of Asia is currently underdeveloped, but it is unlikely to be the only one, for sources relating to epidemics in the Indian subcontinent are relatively abundant and could permit detailed explorations of the relationship among disease, trade, conquest, and environmental change.^45^ The same is true of the[Other P-649] wider Indian Ocean region, identified over twenty years ago as a promising area of study.^46^ But there are no Asian counterparts to the Atlantic studies of Alfred W. Crosby and John R. McNeill—a lacuna that presents a major opportunity to scholars with the requisite skills. The historical coverage of most of Africa prior to 1800 is even more limited, although in this case it is the absence of documentation that is largely to blame.^47^ Only in the north of the continent—in Ethiopia and the Islamic caliphates—is it possible to determine the origins and impact of disease with accuracy.^48^ To date, most of this scholarship has concentrated on ancient and medieval plagues, but recent work on the Ottoman Empire has begun to examine the relationship of disease to environmental change, imperial expansion and commerce in later centuries.^49^ Before long, it may be possible to chart the spread of disease through a large swathe of North Africa and Asia during the early modern period, as well as the ways in which environments changed as a consequence of conquest, war, and commerce.

After 1800, the epidemiological contours of Africa and most other parts of the world are more distinct and the scholarship more abundant. But while the geographical coverage is more even, it remains fragmented. Historians have tended to view disease largely within national or colonial borders, their principal intention being to examine tensions within the body politic. In this sense, the historiography of disease in nineteenth-century Asia and Africa has largely mirrored that of Europe and North America.^50^ Most historians have therefore failed to grasp the broader significance of this period. No less a figure than Emmanuel Le Roy Ladurie dismissed the nineteenth century as a mere footnote to the “exchanges” of 1300–1600, a period which, he insisted, has “no parallel before or[Other P-650] since.”^51^ This generalization holds true only if we confine our attention to the immediate demographic impact of disease. In other respects, the epidemiological upheavals of the “long nineteenth century” were just as significant. The 1800s saw the greatest redistribution of pathogens the world has ever known. Human, animal, and plant diseases circulated in many directions, with enormous social and political ramifications. This global picture usually appears as a dimly illuminated backdrop to a local or national story. It is therefore necessary to think more deeply about the connections between these apparently disparate events.

In the 1790s, the dawn of this new epidemiological era was heralded by a resurgence of yellow fever in the Caribbean. An epidemic subsequently developed in the western Atlantic amidst the tumult of war and revolution.^52^ Spreading northward to the eastern seaboard of America, yellow fever then passed eastward over the Atlantic to reach Europe, seemingly for the first time. For nearly three decades it erupted sporadically but powerfully along the Mediterranean coast, creating havoc in cities such as Cadiz and Barcelona.^53^ After an isolated outbreak on Gibraltar in 1828, the disease was absent from Europe for nearly thirty years, before returning again in the 1850s and 1860s—part of a new wave of disease that engulfed the Atlantic region as a whole.^54^ These pulses of yellow fever were probably related to fluctuations in climate but were sustained by a combination of colonial warfare and trade. The same is true of plague, which spread from the Middle East in the 1800s and 1810s (as far west as Malta) and later of cholera, which radiated out of South Asia from the 1820s.^55^ Other[Other P-651] diseases became prevalent for much the same reasons, their predations aggravated by brutality and dispossession.^56^

As this tumultuous century drew to a close, plague was unleashed from its confines in parts of Asia and North Africa to reach every inhabited continent.^57^ More than any previous pandemic, the resurgence of this ancient disease illustrated the perils of global connections and new forms of transportation such as the railway and steamship. As plague was incapable of sustaining itself for longer than forty days in a confined space such as a ship, “sail-age” pandemics were probably restricted to the Old World. But with the advent of steam power, every country was at risk, and it is no coincidence that the 1890s saw a dramatic escalation in the use of the term “pandemic” to describe widespread infectious diseases. Hitherto, the term had rarely been applied to such epidemics, even in the case of cholera. Many diseases regarded as noncontagious had also been referred to as “pandemic,” as were aspects of lifestyle and morality. By the early twentieth century, however, the modern meaning of the term had become dominant. This was due in large part to plague but also to two major epidemics of influenza: the first in 1889–93 and the second in 1918–19. These were truly global infections, being oblivious of borders and social rank. A mature telegraph network also made possible the reporting of these outbreaks almost as they happened, producing a sense of dread at influenza’s seemingly inexorable progress.^58^

Much has been written about these pandemics—or at least their local manifestations—but the connections between them are seldom considered. Nor have they been seen in relation to other pathogenic exchanges[Other P-652] that arose from the global trade in agricultural commodities. Livestock plagues such as rinderpest, rabies, foot and mouth, and East Coast fever,^59^ as well as plant infections like the blight that caused the Great Irish Famine,^60^ brought hardship and death to millions. Contemporaries saw these phenomena as linked and many regarded the simultaneous spread of cattle plague and cholera as a sign of a world gone awry.^61^ It was the enormous variety of pathogens that distinguished this period from earlier epidemiological crises, as did their diverse points of origin. Although most originated in Asia and Europe, some came from elsewhere, including the Americas. The blight that destroyed the Irish potato crop, for instance, was most likely imported into Europe in shipments of guano from Peru; the diseases and pests that devastated European vineyards at the end of the century were also of American origin. Each of these diseases was related to specific patterns of movement, such as those occasioned by trade, war, economic migration, and religious devotion.^62^[Other P-653]

The most important of these was trade, which expanded massively in scale and scope in the course of the century. To be more specific, most of the century’s pandemics and panzootics were consequences of the reorientation of production from local needs toward international markets.^63^ Imperialism—and British imperialism in particular—provided the catalyst for these changes and gave them a distinctive character and form. But the ramifications of the new world economy were by no means confined to the imperial powers and their colonies.^64^ Around the world, agricultural and industrial production was transformed as states and entrepreneurs began to take advantage of access to a rapidly expanding market. A massive surge in the volume of commodity exports and investments was followed by an unprecedented convergence of prices.^65^ Established industries, farms, and communities were swept away by the force of foreign competition and state-driven development.

We cannot understand the full impact of economic integration unless we consider how environments were changed by their incorporation into a global market. Capturing the complexity of these dynamics in a single narrative is formidably difficult, but one way of doing so is to examine a variety of localities in order to determine how ecosystems were altered as they were drawn into a global web. This is, perhaps, most easily achieved by focusing on an industry or type of economic activity rather than any particular disease. Plantation agriculture is well suited to this, for it illustrates both the global migration of pathogens and their sensitivity to social and ecological conditions. As they developed during the sixteenth and seventeenth centuries, the sugar plantations of the Atlantic came to rely almost exclusively on African slaves. While slavery lingered on in the[Other P-654] American South, it was progressively abolished elsewhere and, by the mid-nineteenth century, most plantations in the Caribbean and Latin America depended on an influx of cheap labor from overseas. The majority came in the form of indentured workers from South Asia and China, and the same was true of the plantations that came to be established in parts of Southeast Asia and tropical Africa.^66^ It was Asia, too, that provided most of the labor to build railways in Africa and North America.

The epidemiological consequences of these migrations have been examined most carefully with respect to the shipment of indentured workers from South Asia to the Caribbean. From these studies, we know that cholera, smallpox, and other diseases often broke out on migrant vessels and that these pathogens were carried to many parts of the world.^67^ But migration within states was equally important. In Russia, the abolition of serfdom in the 1860s resulted in an eastward migration of peasants who seized land from nomadic pastoralists. Deprived of the means of subsistence, many former herders had little option but to work on the farms and mines established by the immigrants. There, they encountered malaria, which had recently been brought into the region. Unable to afford relief in the form of quinine, they suffered terribly from the effects of parasitic infection.^68^ In British India, too, migration stimulated by the growth of a global economy had a profound effect upon the distribution of pathogens. Indentured laborers were transported to tea plantations in Assam and elsewhere in the Northeast by river, bringing with them a variety of infections including those causing leishmaniasis, known locally as kala-azar.^69^[Other P-655] This insect-borne disease quickly became established in monocultural plantations, in which there were relatively few natural predators.

By the 1880s, as the rail link to Calcutta was nearing completion, Assam also experienced what appears to be its first epidemic of cholera. Cholera was common among the laborers who built the railway, and they were often seen as responsible for infecting new areas.^70^ Once introduced, the disease thrived in the insanitary and overcrowded dwellings to be found in the tea gardens. The same was true of the parasitic infection, hookworm. As hookworms are transmitted in feces, the poor sanitation on most plantations meant that the disease became firmly established in many parts of the world. Hookworm diminished the productivity of workers, and it was partly for this reason that it became the target of the Rockefeller Foundation’s first campaigns in disease eradication in the American South and later overseas.^71^ One focus of the RF’s activities was Ceylon (Sri Lanka), hookworm having spread with indentured laborers from South India. Tamil laborers were brought in to work on tea and rubber plantations established by the British, whose brutality and neglect had deterred most of the native Singhalese from seeking employment in them. Conditions in the plantations were so bad that the hookworm infection rate had reached 90 percent by 1916.^72^

Another way of exploring the impact of the global market on disease is to examine its connections with particular commodities. Commercialized rice production, for example, is closely associated with a number of diseases, one of which is malaria. The relationship between rice cultivation and malaria is extremely complex, much depending upon prior exposure of laborers to infection, the stability of previous transmission patterns, and species of mosquito.^73^ Nevertheless, the rising incidence and[Other P-656] prevalence of malaria in many parts of the world was closely related to the production of rice on a large scale, especially where paddies were fed by newly constructed canals.^74^ Rice monoculture also resulted in a decline of natural mosquito predators and consequently in terrible epidemics of malaria, such as those that occurred in the highlands of Madagascar in 1878 and 1895.^75^ The establishment of canal colonies in the Punjab in the late nineteenth century and the irrigation of lands adjacent to the River Indus in the 1920s and 1930s similarly produced a surge in malaria.^76^ Nor were such epidemics confined to the colonies. The commercialization of agriculture and the expansion of large-scale rice cultivation in the American Midwest led to malaria becoming a serious problem in southern Illinois, just as it was beginning to decline in older areas of production in the South.^77^ Being a highly intensive form of agriculture, rice cultivation depended heavily on migrant labor. Workers traveling to new areas of cultivation often brought with them new strains of parasites or were exposed to infection for the first time. Despite growing awareness of the transmission of malaria at the turn of the twentieth century, the profitability of rice cultivation in countries such as Italy stalled attempts to curb exposure by regulating working hours and improving accommodation.^78^ Nor was malaria the only disease associated with commercial rice production. The industrialization of rice farming, especially the process of milling, resulted in the growing prevalence and incidence of the[Other P-657] deficiency disease beriberi. Milling reduced the vitamin-rich husk and in many parts of East, South, and Southeast Asia, populations unable to find the vitamin in other items of diet began to suffer severely.^79^ These examples are intended to show how one might explore long-distance connections without concentrating on the more obvious candidates—the nineteenth century’s great pandemics. Other methods or diseases could serve this purpose equally well. Tuberculosis, for instance, came to prominence as a disease of the industrializing West but later became prevalent elsewhere; not primarily because of infection by Europeans but because of rapid economic change. In Britain, the highest rates of tuberculosis were often in areas in which coal and other forms of mining were the principal sources of employment. The high incidence of diseases such as pneumoconiosis and silicosis probably predisposed miners to develop tubercular infections.^80^ However, “pulmonary consumption,” the nearest contemporary analogue to the disease tuberculosis, was effectively excluded from the purview of the early public health movement in Britain, as were many other diseases that stemmed directly from working conditions. Though it was the principal cause of death, consumption was regarded by Edwin Chadwick as a disease whose causes were too complex to fall within the remit of his enquiry into the health of the laboring poor.^81^ It consequently took many years for tuberculosis to be established as an occupational disease, despite its intimate relationship with industries such as mining.^82^

Cramped and poorly ventilated conditions in factories and mines, as well as in workers’ accommodations, were conducive to the spread of tuberculosis and when these industries relied heavily on migrant labor the problem was magnified. This pattern was evident in South Africa from the 1870s, as mining developed rapidly in Kimberley and on the Rand.^83^[Other P-658] The same was true of India, where disease spread from industrial centers as laborers returned to their villages. In both cases, the contrast with the preindustrial past was striking. Tuberculosis was scarcely acknowledged as a problem affecting Indians until the 1840s but by the end of the century it was rampant in the cotton towns of Western India and the jute manufactories of Bengal. Over the course of the next half century, mortality from tuberculosis and other respiratory diseases rose sharply as deaths from other common infections declined. Nevertheless, it took a long time for the problem to be recognized and it was forced onto the official agenda only through the efforts of Indians trained in public health. The outbreak of the First World War, which saw colonial troops and labor corps mixing with soldiers from the West, caused tuberculosis to spread even more widely. European doctors, whose countries were now experiencing a decline in tuberculosis, began to regard the disease as rite of passage through which all industrializing societies must pass. In their view, it was part of the price to be paid for the advance of civilization.^84^

The circulation of pathogens through the new global economy and, later, during the war presented great difficulties, the most obvious of which was how to regulate the flow of people and commodities without disrupting either the economy or the war effort. As far as labor was concerned, the peacetime balance tilted in favor of movement, as health checks were often cursory and seldom posed much of an obstacle to immigration.^85^ In wartime, the stakes were higher and the controls more stringent.^86^ The case of trade was different again. Quarantine and sanitary embargoes were established means of controlling the spread of infectious disease, but nations were regularly embroiled in disputes over the legitimacy of such measures, some of which devastated their economies.^87^ By the start[Other P-659] of the twentieth century, however, a new strategy had been devised, relying more on surveillance and preemption than the prohibition of movement.^88^ This was one of the tasks of newly established organizations such as the Pan American Health Organization and the Office Internationale d’Hygiène Publique.^89^ However, agricultural diseases remained a problem, for the measures taken to prevent their spread had a greater impact on the economies of afflicted nations. The depression of the late nineteenth century and increasing transportation of bulky commodities such as animals, meat, and grain sparked trade wars in which the supposed risk of infection figured prominently.^90^

A more difficult question was how to temper development in the interests of public health. The balance was struck differently in different places: in some, considerations of health were subsumed by the pursuit of profit, especially where labor was bonded, cheap, and plentiful.^91^ But usually health and productivity were not mutually exclusive. Disease affected productivity after all, and this was increasingly recognized during the twentieth century as more attention was paid to the health of the workforce.^92^ But the relationship between health and productivity often depended on racial considerations and on the extent to which industries faced labor shortages. It was not until the 1930s that South African mining officials became seriously concerned about the health of their African[Other P-660] workers, for example. Only at that point did they began to realize that the supply of black labor was finite, whereas recognition of the need to protect the health of white workers, whose numbers were more restricted, came considerably earlier.^93^

Before moving on to examine medicine, I wish to reiterate the main point of the discussion so far: that thinking globally about disease means more than considering the movement of pathogens or even relative immunity. Disease is a powerful agent of change, but it never operates in a vacuum and to regard it is a force *of* globalization is simplistic.^94^ It is necessary to identify the dynamics that forge pathogenic connections and that simultaneously transform the social and natural ecologies into which pathogens and vectors are introduced. Only in this way can we account for the differential impact of disease and its longer term consequences. It is important to remember that the nineteenth century saw the emergence of markedly different regimes of mortality, despite the global circulation of pathogens. From the middle of the century, public health interventions and improved nutrition resulted in falling death rates in many Western countries, while mortality rates in their colonies remained stable or increased.^95^ The legacy of this divergence is, of course, still much in evidence.^96^ To put it another way, those countries and social classes that were able to exploit global connections flourished and benefitted from advanced systems of public health and health care. This included not only Europe and North America but also some of their dominions, such as New Zealand, which grew rich as a result of long-distance trade in agricultural commodities. By the early 1900s, New Zealand was well on the way to becoming a social laboratory that others would emulate.^97^ But those who[Other P-661] were driven into penury or forced to seek work as migrant laborers saw a decrease in their standard of living and state of health. These refugees from forest and field—concentrated in the European colonies—were the losers of the new global system, much as their counterparts today.^98^

## Medicine

One characteristic of historical scholarship over the past two decades has been its sensitivity to different voices and experiences. From this standpoint, the rise of Western medicine is not the only story worth telling, and to write a global history of medicine solely from that perspective would be unacceptable to many.^99^ This remains one of the greatest challenges facing our field, for general histories of medicine have concentrated almost exclusively on the Western medical tradition.^100^ Although Henry Sigerist devoted a large section of the second volume of his unfinished multivolume history to ancient India and Persia, this appears to have been a prelude to the early history of the Western tradition, much as in the earlier work of Garrison, for example.^101^ Conscious of the neglect of other traditions, Roy Porter devoted two chapters to Indian and Chinese medicine respectively in *The Greatest Benefit to Mankind*. Yet these chapters sit rather uneasily with the others, which are devoted to Western medicine through the ages and in various manifestations.^102^

It is probably impossible to give equal weight to all medical traditions in a single volume, and to do so would require a breadth of scholarship far beyond the majority of historians. Such an ambitious undertaking might be feasible as a collective enterprise, but a more expedient approach for the lone scholar would be to examine interactions between medical cultures over a shorter time span. Even then, one encounters a multitude of[Other P-662] problems, not the least of which is deciding when to begin. Dates that are significant for one tradition are not necessarily significant for another. Deciding where to begin is no easier. As late as 1800, Western medicine was not as widely diffused as *tibb*, a descendent of ancient Greco-Roman medicine that was practiced widely throughout the Islamic world.^103^ A global history of modern medicine might just as well commence in the courts of Constantinople or Bukhara as in the medical schools of Europe.

My task in this article is fortunately less ambitious, for it is to examine the impact on health and medicine of the global market. Until the turn of the twentieth century, this was most clearly evident in the rise of Western medicine, and any general study has to acknowledge that fact. Other medical traditions lacked the expansionist and hegemonic aspirations of Western medicine and even the most widely disseminated—tibb—adapted to those localities into which it had spread. Other medical cultures (such as ayurveda) made a virtue of locality and of their organic relationship with places and peoples. Western medicine was different because its truth claims were universal and because it was bound closely to ideas of progress and reform.

Modern “Western” medicine (the term was not widely used until the twentieth century) is normally thought to have emerged in Europe, or more specifically in Paris, at the end of the eighteenth century.^104^ However, its origins were far more diverse and are to be found outside Europe as well as within it. The expansion of long-distance commerce fostered an empirical sensibility in Europe and its Asian and American colonies, and this contributed to the emergence of new forms of knowledge.^105^ European merchants began to cultivate and exploit a cosmopolitan trade[Other P-663] in medicaments as well as actively engaging in a search for new botanical remedies. Not all of these items were adopted for use in Europe, but the opening of Asia and the conquest of the Americas enabled Europe’s therapeutic repertoire to expand.^106^ The colonies also afforded great opportunities for Europeans who wished to investigate diseases and their relationship to the natural world, as well as to view their appearances postmortem. Similar opportunities were not available in Europe until the early nineteenth century, but ideas and practices from the colonies began to filter through to Europe before that, contributing to a growing clamor for reform.^107^ Modern (Western) medicine, then, was spread through not only channels of global interaction, but was also, in some measure, their offspring. Centers of innovation in Europe existed in a dynamic relationship with one another and with the colonies, linked by networks of long-distance trade, correspondence, and a plethora of new medical journals. Medicaments and dried plant specimens (sometimes accompanied by relevant literature) followed routes that originated within but ultimately expanded beyond imperial networks.^108^ We are only just becoming aware of the scope and importance of these connections, but they suggest the need to revise conventional, Eurocentric accounts of the rise of modern medicine.

After modern “Western” medicine became established at the turn of the nineteenth century, its champions were at great pains to distance themselves from vestiges of “superstition” in their own past and what they perceived as such in other traditions. Their assertiveness reflected[Other P-664] newfound confidence in the experimental sciences (which had begun to have an impact on medicine), but it had an ideological dimension, too, and this acquired its sharpest definition outside of Europe and North America. It was here, from the 1820s and 1830s, that medicine, like other forms of science, was projected as a civilizing influence, attaining greater prestige against a backdrop of “ignorance” and “unthinking tradition.”^109^ But Western medicine was far from monolithic. One obvious difference was between missionary medicine—with its conflation of science and faith—and the more systematized medicine practiced in major hospitals.^110^ Even within state institutions there were significant variations in the practice and character of Western medicine as experienced in day-to-day encounters in small dispensaries and clinics.^111^ To varying degrees it was adapted and taken up by non-Western peoples, reflecting their preoccupations and cultural preferences as much as government policies.^112^ Furthermore, hospitals and medical schools in parts of Asia, Africa, and the Americas were pioneering concepts and techniques that became popular elsewhere, including established centers of learning in Europe. During the nineteenth and early twentieth centuries, the crossover was most evident in the fields of bacteriology, parasitology, and public health. Much of the pioneering research on malaria and cholera—still very much European diseases—was done using personnel and medical facilities in the colonies.^113^ The colonies also provided fertile soil for innovation in psychiatric medicine, and some practices devised overseas were later adopted in Europe.^114^[Other P-665]

By the end of the nineteenth century, Western medicine was thoroughly international. There were flows and counterflows of ideas, people, and practices, following the contours of the new global economy. Some took familiar pathways, but others—like those resulting in international medical congresses—were novel and exhibited a strong commitment to progress.^115^ But why was it that Western medicine came to be so widely disseminated and highly valued?

For a long time, the answer appeared to be simple: Western medicine was self-evidently superior, and people came to prefer it to less effective local remedies. But while certain drugs and procedures such as vaccination against smallpox had enormous potential, it took some time for this to be realized due to technical and other practical constraints, let alone the cultural and political obstacles that impeded their introduction.^116^ Some historians have therefore sought an explanation in the symbolic role of Western medicine in modernizing states, particularly colonial ones. Backed by the power and resources of government, they argue, Western medicine came to enjoy immense prestige while other traditions were relegated to a position of inferiority.^117^ There is some truth in this, but one ought not to push the argument too far. Many indigenous traditions were able to survive and even to thrive throughout the nineteenth century.^118^ This was true not only of colonial societies but also of some independent countries that regarded Western medicine as a tool of modernization. While older medical traditions were obliterated in Japan, for instance,^119^ they were fostered alongside newer ones in the Ottoman Empire, Qajar Iran, and some of the “Princely States” of India.^120^ Western medicine[Other P-666] ultimately gained the upper hand in most of these countries because its impact was magnified by the near ubiquitous presence of Western pharmaceuticals, some marketed globally, others locally or regionally. In the late nineteenth century, most countries were transformed by the economic forces described in the last two sections. The commercialization of agriculture, industrialization, urbanization, and labor migration placed many more people in contact with Western medicine and its products. Medicines and medical services were widely advertised and available in shops and in the workplace through numerous vendors and practitioners. This encouraged many people to experiment with a range of therapeutic alternatives to traditional remedies. “Doctors” and druggists without formal qualifications traded in streets and bazaars, offering Western medicine at a price that many could afford. Those who had invested considerable time and money in a medical education were acutely aware that they were being undercut by cheaper, unlicensed competitors and began to demand the registration of medical practitioners. Legislation like that which had been enacted in Europe and North America was increasingly passed in other parts of the world. By 1914, for example, all provinces of British India had introduced a Medical Registration Act modeled on the one passed in Britain in 1858.^121^

This legislation prevented unqualified people from calling themselves doctors, but it did little to curb the activities of irregular practitioners who peddled a bewildering array of patent medicines. Although most of these remedies offered little more than hope, there were enough reasonably effective drugs on the market (quinine tablets for example) to sustain the growing belief in the efficacy of Western medicine. The process by which this new medicine came to be embedded in Asian and African countries was not dissimilar to what had happened in Europe a century earlier. Then, an influx of exotic drugs such as fever bark (from which quinine was later synthesized) and newly discovered indigenous ones such as willow bark (from which Aspirin was later derived) boosted confidence in medicine while fueling a burgeoning market in patent remedies. That the boom in pharmaceuticals and advertising paved the way for the dominance of Western medicine is also indicated by the response of many traditional healers. They recognized that they could compete effectively with Western medicine only by employing similar tactics: by commodifying their drugs and taking advantage of new print media to advertise their products. As we shall see in the final section, practitioners[Other P-667] of ayurveda, tibb, and Chinese medicine used these methods not only to survive but ultimately to extend their influence beyond local markets.^122^

## Health

At first sight, health would appear to be an unpromising subject for global history. Its subjective nature lends itself to the investigation of difference rather than shared or connected experiences. And yet, it is possible to think globally about health in a way that acknowledges difference and change over time. Over the past two hundred years, conceptions of health have been transformed as a result of insights from immunology, physiology, psychology, and other sciences. Health has come to be regarded not simply as the absence of disease but as a state of optimal fitness or well-being. This conception of health has provided scope for self-realization, but it has also been deliberately encouraged and manipulated.^123^ In the early twentieth century, a cluster of ideas began to form in which health was linked to civic obligations. Citizens were encouraged to think about their collective and national responsibilities, and many aspects of their lives came to be regulated in the name of public health.^124^ Beginning in the West, these ideas soon spread to other parts of the world. New institutions such as medical colleges, modern hospitals, and dispensaries played an important part in this, but no less important were the media through which the public sphere was constituted. Newspapers and journals of all types and in myriad languages popularized Western notions of health and hygiene through their articles, editorials, and advertisements.[Other P-668]

The pills and tonics advertised in the pages of popular journals were the material artifacts of a new culture of health that blended imported, Western perspectives with local ones.^125^ They were accompanied by a variety of soaps, cosmetics, and cleaning materials, whose implicit messages were made explicit through formal education and propaganda in schools, films, posters, and lectures.^126^ Indeed, the notion that health was a social obligation was among the most important aspects of modernity as it was experienced, with subtle variations, in different parts of the world. One is accustomed to seeing these things as national or colonial phenomena, but there was a substantial degree of convergence between countries despite their different cultures and political systems. This “hygienic modernity” had colonial, liberal, socialist, and fascist inflections,^127^ but these were variations on a theme, and the variations are arguably less impressive than the similarities. Imperial competition, social Darwinism, economic hardship, and the threat or actuality of armed conflict placed a premium on bodily and mental efficiency. This began before birth and lasted throughout an individual’s productive life.^128^ A new view of health, which sought to enhance physical and mental performance, became firmly entrenched as societies came to be organized in the service of mass manufacture and warfare.^129^ Such imperatives were not confined to Western nations or[Other P-669] even to the cadres that governed their colonies. The educated elites of many Asian and African countries also came to view health as an index of social fitness, and its promotion was championed as a means of racial and national improvement.^130^ As in the West, growing consciousness of health was connected with dietary faddism, physical training, eugenics, and campaigns for moral and mental improvement. Decades of colonial critique had been internalized, and colonized peoples strove to overcome and surpass the physical standards set by their masters. In some cases, most obviously in Japan, the cultivation of health was seen as the path to modernity and imperial dominance.^131^

Around the world, the promotion of health was embraced by the state and modernizing reformers. As taught in schools and inculcated in institutions such as the armed forces, hygiene conveyed the rudiments of Western medical science and led to rising demand for products legitimated by bacteriological conceptions of disease.^132^ However, the penetration of bacteriological and physiological models of health, as well as those derived from psychology, did not completely displace older ideas based on ancient ideas of balance or, indeed, religious principles. In some cases, elements of modern of science were blended with insights from non-Western medical traditions, religions, and philosophies. Paradoxically, this became evident at the same time as modernist conceptions of health were articulated with most conviction. During the 1930s, for example, some individuals in Western countries began to promote alternative visions of health, some of which were explicitly or implicitly critical of modernity and its tendency to fragment human consciousness.^133^ One aspect of this was the revival of[Other P-670] holistic conceptions of health, some of which were closely linked to dietary regimes like vegetarianism and whole-food consumption, as well as the organic movement in agriculture.^134^ In each colony and nation-state the rendering of science and its blending with indigenous cultural traditions was different, as were the political circumstances in which strictures on health emerged. Yet, universal principles were increasingly evident: the result not only of a particular vision of industrial modernity but of the increasingly important role played by international organizations such as the League of Nations Health Organization and the Rockefeller Foundation. Although these bodies were intended primarily to manage populations and the effects of economic change, they raised aspirations and set global standards by which public health and its outcomes could be judged. In the case of the Rockefeller Foundation, they also exported a model of public health that included the establishment of urban and rural health units, demonstration projects, and education in hygiene from school to university. Although the RF had little lasting impact on British India, for example, its programs were implemented widely elsewhere, particularly where they complemented local initiatives and nationalist projects of regeneration.^135^ Public health was progressively internationalized, its focus transcending purely colonial concerns.^136^

By the 1930s differences in health indicators could be measured fairly precisely due to the centralization of mortality data in the League of Nations and bodies such as the Pan American Health Organization.^137^ As a result, something approaching a global consciousness of health emerged,[Other P-671] with international comparisons—often involving the Soviet Union as a paragon of the new collective ideal—figuring prominently. While countries were seen to be at different stages of development and to face different challenges, there was, as Henry Sigerist put it, a “human solidarity” in matters of health that could not be easily disregarded.^138^ This was to acquire more substantial form in 1948, when health was listed as a human right in the United Nations Declaration and the idea grew that states had important obligations to ensure the health of their citizens. However, the internationalism of the immediate postwar era began to evaporate as relations between the West and the communist powers deteriorated. The notion that health was a human right was also criticized in some quarters as vague and impossible to define.

Nevertheless, most affluent nations continued to spend generously on health and to promote its development in poorer countries through foreign aid. Thus, the two superpowers—the United States and the Soviet Union—were major players in the most high-profile public health intervention of the postwar era: the World Health Organization’s campaign to eradicate smallpox. The latter began in 1959 following the initiative of the Soviet Union’s deputy minister of health and intensified from the mid-1960s, under American leadership.^139^ Such campaigns reflected the technological optimism that had emerged in the preceding decades, but they were also an expression of political will, on the part of both national governments and the international community represented by the United Nations and its agencies. The United States and the Soviet Union also gave technical and financial assistance to international health campaigns in the hope that it would improve their standing in countries regarded as ideological battlegrounds. This proved beneficial or divisive depending on the context. Indeed, campaigns such as those waged against smallpox and malaria in the 1960s and 1970s highlighted many of the problems that would become increasingly evident in the years ahead; namely, tensions between local and international agencies and the tendency for high-profile, “vertical” public health campaigns to attract resources from other services.^140^ As we shall see in the next section, these problems were[Other P-672] aggravated by the proliferation of actors in the field of global health and by the diminishing power of the state.

## The Global Present

In this final section of this article I consider some contemporary issues that would benefit from an historical perspective, starting with the most obvious consequence of globalization—pandemic disease. This aspect of contemporary globalization has received more attention from historians of medicine than any other, many books and articles having been written on HIV/AIDS and SARS in recent years. However, nearly all of these studies examine disease in the context of particular cities or nation-states; only a few offer a global or comparative perspective.^141^ Such a perspective is necessary, to understand not only the causes of pandemics but also their legacy. The pandemics of the past few decades have already left a mark on our consciousness and this is particularly true of AIDS, which the historian Allan Brandt regards as the catalyst to new ways of thinking about public health. It was AIDS, he argues, that gave birth to the new field of “global health,” now widely represented in university curriculums and in government programs around the world. Brandt sees clear differences between global health and the era of international health that preceded it. In particular, he points to the much greater emphasis that the global health movement places on human rights, the more active role envisaged for recipients of health care, and the integration of public health with clinical medicine. But these “complementary innovations,” as Brandt terms them, jostle with some very different conceptions of what global health is or ought to be.^142^ Global health manifests differently according to the problem identified, while the objectives of its agents vary considerably.^143^

The tensions within what would become the global health movement were present at its inception. While activists demanded respect and adequate treatment for the victims of AIDS, governments resourced AIDS programs largely for other reasons. Chief among these were concerns over security; that is, the protection of national interests. The devastation[Other P-673] caused by AIDS in sub-Saharan Africa led intelligence agencies to contemplate the implications of the pandemic for the stability of allied and strategically important states.^144^ Disease was not the sole source of these anxieties, but it came to embody them, whether it was the volatility of the globalized economy or concerns over terrorism.^145^ Today, governments continue to view global health initiatives in similar ways.^146^ While they stress the need to work with external agencies to achieve common objectives, their decisions are guided chiefly by national interests. The aspiration of “health for all” is secondary, if not always incompatible with the first.^147^

The aims of different global health agencies do not necessarily contradict one another, but there is certainly some tension between them. At the dawn of the new millennium, fear of new infections—originating mostly in tropical countries—led some in the West to demand tighter controls on the movement of peoples and commodities from areas deemed to have a higher risk of infectious disease.^148^ On the whole, they were left frustrated, but more emphasis came to be placed on epidemiological surveillance and containment, especially after the SARS pandemic of 2003. Although it killed relatively few people by contrast with most other pandemic diseases (8,422 recorded cases and 916 deaths), the uncertainty surrounding SARS caused great alarm and threatened for a time to destabilize the global economy.^149^ The response to SARS, coming in the wake of the[Other P-674] terrorist attacks of September 2001, was infused with the rhetoric of the War on Terror. Nations that failed to observe the new protocols of “germ governance” were seen as little better than states that sponsored terrorism.^150^ There was a sense that the world had become a more dangerous place, in which nation-states would struggle to protect their sovereignty and their borders. The response to SARS invariably took the form of quarantine and isolation, violation of which, in some countries, was severely punished.^151^ While there was general acceptance that such measures were desirable, some states were accused of overreacting and of unacceptable infringements of human rights. In Taipei, for example, the city’s homeless population was corralled and healthy people were placed in quarantine alongside those who were infected.^152^ This draconian response was probably intended to reassure the international community, for states that failed to meet international expectations faced censure and a catastrophic loss of business and investor confidence.^153^

In all these senses, SARS was typical of the majority of epidemic or pandemic diseases. AIDS, by contrast, was a slowly unfolding tragedy rather than a time-limited event or sequence of events.^154^ It was therefore SARS rather than AIDS that provided the model for pandemic control in the coming decade. The main focus of these concerns was influenza or influenza-like diseases, which were capable of spreading rapidly in an age of mass air transportation and of engendering a panic that could destabilize the global economy. These threats were (and are) real enough, but they also reflect the volatility of financial markets and other anxieties arising from globalization. These fears were stoked by estimates of mortality that were invariably inflated, sometimes egregiously so. This was not solely because public health officials played to the media but because the[Other P-675] methods they employed were flawed. Influenza is perhaps the most protean of infections, and most models designed to predict its behavior have proved unsatisfactory.^155^ But predictions—even inaccurate ones—have their uses, and governments have taken advantage of the fear generated by pandemics to justify measures normally considered unacceptable. During the H1N1 pandemic in 2009, for example, many countries imposed a ban on pork imports from North America despite a declaration from the WHO that such produce was safe. This prompted allegations of sanitary protectionism—a familiar refrain since the dismantling of formal tariff barriers and the creation of the World Trade Organization in 1995.^156^ Yet, sanitary protectionism is far from being a “weapon of the weak.” The states that have most frequently taken advantage of international law are among the richest and most powerful. The losers tend to be those that rely on export earnings to improve the health and prosperity of their people.

The fragmented response to H1N1 contrasts starkly with the consensual, internationalist rhetoric of the global health movement. Though pandemics are universal risks, they continue to elicit nationalistic responses,^157^ and the trend toward global governance, which some claimed to discern in the response to SARS,^158^ has stalled. Repeated demands to “securitize” health^159^—if enacted—are likely to exacerbate these problems, for, while security is clearly a “public good,”^160^ it is generally invoked in defense of particular interests. As we have seen, this is especially true when sanitary concerns impinge on those of commerce. Most nations employ specialist lawyers and scientists who are engaged to calculate the sanitary risks posed by the trade in certain commodities. Though all appeal to “science” as the arbiter of commercial and other sanitary disputes, there is no objective standard against which such assessments can be made.^161^ Each is[Other P-676] open to interpretation and endless challenges result. Calculations of risk also shape other aspects of pandemic prevention. Most countries have national risk registers in which pandemics figure prominently, while insurance companies make their own influential assessments. The company Maplecroft, for instance, produces an annual Influenza Pandemic Risk Index that “enables governments, intergovernmental organizations and businesses to identify potential risks to populations and supply chains.” Countries are ranked and classified according to different risk levels along three indices: the risk of the emergence of new strains, the risk of spread, and their capacity to contain an epidemic.^162^

Risk assessments are unavoidable and, in many respects, beneficial features of public health. However, we ought not to assume that they are objective statements of reality. It is vital to understand how risks are calculated and who stands to gain from such assessments. Individuals, social groups and even nations can be pressurized into altering their behavior in conformity with these calculations—whether by the dictates of insurers or through fear of legal redress.^163^ But the most pressing need is to understand the process whereby risk calculations result in risk management. All manner of assumptions and interests come into play as communities or organizations decide how to interpret and act upon the risk assessments they receive. It is also important to reflect upon the ways in which risk assessments affect inter-personal relationships. Humanitarian and cosmopolitan sentiments can easily evaporate once risk groups have been identified. Evaluations of risk also provide a more atomized and individualistic basis for public health than ideas of civic duty, whether they be Renaissance notions of the “common good” or modern conceptions of “social citizenship.” The latter are essentially collective principles and entail mutual obligations. Risk assessments can be utilized in the service of such ideals, but they tend to pull in the opposite direction. This is clearly the case with vaccination, the uptake of which has dropped in many high-income countries, especially in wealthy areas. Parents make choices based solely on the risks and benefits of vaccination for their own children, as opposed to notions of collective responsibility. Herd immunity has come to be seen not so much as a social good but as a factor in individual decision making.^164^[Other P-677]

Risk relationships may be no better or worse than earlier foundations of public health, but they are different and need to be reckoned with. Faced with a multiplicity of risk factors, national governments, charities, NGOs, and global institutions are left to decide which to prioritize and how to act on them. Since the appearance of SARS, there has been a clear preference to deal with the risk of pandemic diseases by tracking their emergence and spread, with the aim of containing outbreaks or buying valuable time. Calls for more and better surveillance therefore persist.^165^ But it is improbable that even the most sophisticated systems of surveillance will enable the containment of all diseases. Moreover, this response deals with only one element of risk—the risk of transmission; the risk of the emergence of new diseases or strains receives considerably less attention.

By far the most important risk factor in this sense is humanity’s changing relationship with other species. All periods of global integration have left their mark on these relationships and the past few decades are no exception. Deforestation—largely as a consequence of logging for the global market—has brought human beings into contact with a wider range of infections, as humans invade the wild habitats of other animals. Ebola, Nipah virus, hantavirus, Rocky Mountain spotted fever, and many other diseases have “emerged” and spread as a result of forest clearance, as well as the increasing recreational use of woodland and other wilderness areas.^166^ The trade in animals as pets and bush meat has exacerbated this trend, most obviously in the case of SARS and Ebola.^167^

Notions of “emerging” and “reemerging” infections sometimes conceal deeply held prejudices about other nations, especially their sanitary habits and modes of governance.^168^ But there can be no doubt that the global disease environment is changing. The rapid growth of cities such as Kinshasa and Chongqing reflects the economic advantages to be gained from concentrating people and resources, but a penalty has usually to be paid. Dense populations allow diseases to circulate and mutate more quickly. Poorly planned construction provides ample breeding sites for mosquitoes carrying diseases such as dengue and malaria. Burgeoning populations outstrip the supply of wholesome water. Most worrying of all, perhaps, are[Other P-678] the consequences of feeding such conurbations. In many parts of Asia, rapid urbanization is the chief driver of intensive animal production, as it was in the West a century or so before. As well as affording greater opportunities for the mutation of viral diseases such as influenza,^169^ the subtherapeutic use of antibiotics in intensive farming has been linked to bacterial resistance, for example, to the drug tetracycline. The problem of intensive livestock production is widely acknowledged by bodies such as the WHO and the WTO.^170^ But there is little they can do in the face of huge variations in national legislation. In view of the powerful interests involved, and the desire to maintain or secure competitive advantage, the prospect of concerted global action appears dim.

So far, I have discussed globalization in relation to pandemic diseases and that is largely because the epidemiological consequences of integration are generally seen in that way. However, there is growing recognition that changing lifestyles, which are directly and indirectly linked to globalization, are transforming patterns of morbidity in both highly developed and low-income countries. Globalization has lifted millions from poverty and has contributed in many instances to the improvement of health infrastructure and the provision of vital utilities such as clean water. It has also enabled governments in developing countries to afford more medicines and vaccines, thereby reducing deaths from easily preventable and curable diseases. These results have been most spectacular in nations such as Bangladesh that have high levels of civic activism, female education, and state involvement in health care. As a result, global mortality from the majority of infectious diseases—with the exception HIV/AIDS—is falling. Causing around 25 percent of deaths worldwide in 1998, infectious diseases were responsible for less than 16 percent of global mortality in 2010.^171^ Global life expectancy has risen to sixty-six years and is expected to reach seventy-three by 2025.^172^[Other P-679]

However, longevity and prosperity have created new problems. An aging population is more likely to suffer degenerative diseases such as dementia, and this is placing an enormous burden on even the richest nations. Rising incomes in developing countries have also brought an increase in alcohol- and tobacco-related diseases. Global mortality from tobacco-related illness, for example, is projected to increase to around ten million per year by 2030. The costs for the countries that are most affected—principally China and India—will be staggering, not to mention the impact on productivity.^173^ The effects of these diseases are compounded by those arising from obesity.^174^ Although conditions such as diabetes and cancer have been rising in developing countries for decades,^175^ the pace of dietary change over the past twenty years has been extraordinary. In China, the consumption of animal products increased 40 percent between 1989 and 1997 and that of “fast food” doubled between 1999 and 2007.^176^ Traditional diets that are relatively high in vegetables, fruits, and whole grains (allowing for local cultural differences) have been replaced by those high in saturated fats and refined carbohydrates. These rapid changes in food culture have been fueled by some of the indirect effects of globalization such as economic insecurity and urbanization, while their detrimental effects on human health have been exacerbated by more sedentary working patterns and increasing reliance on motor transport.

It is presently unclear whether these harmful trends can be countered, but the problem appears rather differently in different countries. In rapidly developing countries there are stark contrasts between the mortality and morbidity profiles of the new middle class and the very poor, many of whom still die as a result of exposure to infectious diseases, accidents, and violence.^177^ Likewise, diseases of affluence in developing countries are often the diseases of poverty in high-income countries. Obesity, cardiovascular disease, and type 2 diabetes are usually to be found among[Other P-680] those unable to reap the benefits of globalization: the poorly educated and unskilled who have suffered economically and psychologically from the decline of traditional heavy industries. In many affluent and middle-income countries there is also a third group of persons who present a complicated mixture of problems: economic migrants. Low-skilled migrant workers are not always from foreign countries but are often first-generation immigrants from rural areas. Like their foreign counterparts, they tend to lack basic rights, including the right to health care and health insurance; their hours of work are long and their job security low. They typically suffer from health problems that show the consequences of new dietary habits (e.g., diabetes) alongside those associated with poor housing and working conditions (e.g., water-borne diseases, respiratory disease, exposure to environmental toxins, and physical injury).

Migrant workers—whose status is sometimes that of illegal immigrants—also exhibit a range of mental health problems caused by overwork, poverty, abuse, and deracination. They share these problems with many persons who arrive in countries of all types as refugees following famine, war, and natural disasters. As Zygmunt Bauman has remarked, the treatment of these groups resembles that of “vagabonds” and other persons displaced during previous economic and political upheavals.^178^ Like their historical counterparts, the marginal status of migrant workers and refugees has also been “fixed” by the stigma of infectious diseases such as tuberculosis and accusations of mental illness.^179^ It is, however, difficult to be sure of the impact of globalization on mental health. For some, globalization may provide a release from social and cultural restraints, whereas for others economic insecurity results in depression and anxiety. What is certain, however, is that current problems of mental health, like those of physical health, cannot be understood independently of the global forces that govern so many aspects of our lives.^180^

The effects of globalization have been equally apparent in the field of health care, most obviously in a shift from public to private provision. The mid-twentieth century saw the idealization of the state as a provider of health care and in most countries its contribution continued to grow[Other P-681] for decades. Western social democracies expanded the range of health care available to the public, while some authoritarian regimes like South Korea under Park Chung-hee favored state-funded health insurance as a way of co-opting and mobilizing the population for nationalist ends.^181^ The same was true of many countries in what used to be called the “Third World.” There, attention shifted from disease-specific campaigns to primary health care. The Soviet Union was one of the leading lights in this transition, hosting the conference that produced the famous 1978 Alma-Ata Declaration of “Health for All by 2000.”^182^ Communist China offered a different model of health care in the form of its barefoot doctor program, which provided an alternative to more hierarchical and technically oriented forms of intervention.^183^

By the 1980s, however, a radically different form of consensus was emerging, closely aligned with the ascent of neoliberal ideology. One of the consequences of the “Washington Consensus” was the scaling back of state-funded health care and the emergence of market-driven reforms. Neoliberalism was sustained by the growing belief that the state impeded efficiency and prevented formerly dominant nations from competing in a global market with states that had lower taxes and production costs. However, the effects of neoliberal thinking were most immediately apparent in low-income countries that were forced to cut back on state expenditure as a condition for financial assistance. As the International Monetary Fund’s structural adjustment programs began to bite into state expenditure, health inequalities became more visible in many of the poorest parts of the world.^184^ Other health care providers including private companies, NGOs, charitable foundations, and missionaries began to take a prominent role.^185^ The work of these institutions has often been impressive, but concern over their accountability remains.^186^ Neoliberal policies also became the norm in the West, even in those countries that retained state-funded[Other P-682] systems of health care. So-called public-private partnerships were promoted vigorously by governments of all complexions. Beginning in Australia in the 1980s, but later taken up enthusiastically in the United Kingdom and other European countries, PPPs seemed to offer substantial savings. The results, however, were mixed, and the cost of such schemes was often massively underestimated.^187^

Privatization has been driven by rising costs as much as by political ideology. As Roy Porter put it some years ago, “the healthier western society becomes, the more medicine it craves.”^188^ Demand for new drugs and other forms of treatment means that the cost of health care has escalated, pharmaceutical companies having an incentive to raise prices to the maximum the market will bear. In countries with substantially state-funded health services, access to new drugs is normally regulated by bodies that assess value for money and clinical efficacy, so the cost of health care has risen more slowly than in countries in which the private-sector dominates. In 2009, the United States, with its predominantly private system of health care, spent 17.6 percent of gross domestic product on health, whereas the United Kingdom, with its free universal health care, spent only 9.8 percent, with no obvious difference in outcomes.^189^

However, the insatiable demand for new medicines is no longer confined to the West, if it ever was. Medical consumerism is now a global phenomenon, and its appetites are sharpened by a cosmopolitan market in goods and services. Western medicine has to compete with other forms of healing such as traditional Chinese medicine and ayurveda, both of which have found niches in a global market. Interest in these and other “alternative” medicines rose sharply from the 1960s, as confidence in Western medicine began to decline following a series of scandals (thalidomide being the most obvious) and concerns over iatrogenic disease. Non-Western therapies were in tune with a counterculture characterized by individualism, environmentalism, and feminism; Western medicine, by contrast, seemed increasingly anonymous and in league with “Big Pharma.” Disenchantment brought a surge of interest in “holistic” forms of healing and pharmaceutical products which touted their exotic or wholesome origins.^190^[Other P-683]

Ironically, the globalization of these products (and the medical traditions which purportedly spawned them) was possible only because they emulated many features of Western medicine and pharmaceutical manufacture. Formerly diverse practices were welded together into traditions “recovered” or perhaps invented in order to meet the need for national systems of medicine in the wake of political independence. Granting equal status to “indigenous” medicines alongside that of the West also meant imposing similar qualifications and standards for practice and training.^191^ This was not uncontroversial but it allowed entities such as traditional Chinese medicine to take a concrete form and this gave them greater prominence internationally. The tendency toward uniformity has been most evident in the pharmaceutical sector, however. Although they have been marketed as alternatives to mass-produced, synthetic pharmaceuticals, many so-called traditional medicines are manufactured industrially and sold in much the same way as their Western counterparts.^192^

Purveyors of these pharmaceuticals have created many global brands but practitioners of non-Western medicine have also had to adapt to local cultures. This is the case with traditional Chinese medicine in East Africa, where, despite the training of African practitioners in Chinese institutions, local expectations have led to significant modifications of practice. One might consider this to be an example of “glocalization” rather than globalization pure and simple.^193^ Other medical “traditions” have also adapted to new locations and different constituencies, as is evident in the case of ayurveda, in which at least three distinct varieties can now be discerned.^194^ In acknowledgment of this complex mixture of diversity and global branding, some scholars have used the term “neotraditionalism” to describe the deployment of “tradition” to legitimate practices which are new.^195^ Being very much a product of globalization, neotraditional[Other P-684] medicine is deterritorialized and carries little of the nationalistic and, on occasion, racial baggage of earlier times.

While some forms of traditional medicine have taken advantage of globalization, others—such as Korean medicine—have yet to establish themselves internationally. Nor should we exaggerate the status that even the most successful forms of non-Western medicine currently enjoy. Some—like traditional Chinese medicine—are recognized in programs of integrated medicine that combine TCM and other “traditional” therapies with biomedical ones. In some Chinese hospitals, integration of Western, TCM and ethnic minority medicines works extremely well, assisted by the fact that practitioners of all systems have identical pay scales.^196^ But while some Western universities have schools of medicine, it is not yet widely practiced beyond Asia and is still looked upon skeptically by many. IM also affords less equality to traditional medicines than might be supposed, for non-Western therapies—which were originally formulated to meet the needs of individual patients—are appraised by randomized control trials and other methods that take no account of such variations.^197^ Far from being a savior of traditional medicines, evidence-based medicine has effectively subordinated them, forcing them to accept the validity of criteria they can never hope to meet.

Biomedicine therefore remains in the ascendant in most countries and is becoming more powerful in some which had vibrant traditions of their own. Western medicine is now seen as a lifestyle choice—just as alternative medicines are for many people in the West—affirming modernity and membership of a global community. It also provides quick and effective remedies that are well suited to the pace of life in a modern, globalized world. As a result of these and other local factors, practitioners of traditional medicine in some countries have seen their status fall. Traditional medical schools in South Korea, which used to attract the best students from high school, now appear to be struggling to fill places as star pupils flock to colleges of Western medicine.^198^ Likewise, foreign visitors are as likely to come to China or India for treatment in Western hospitals as to seek alternatives to such remedies.^199^ But millions in the world’s poorest[Other P-685] countries are unable to afford either the services offered by practitioners of Western medicine or those of physicians trained in the great traditions of Asia. Their medical choices are affected by globalization but not in any clear or easily generalizable way, often resorting to unlicensed practitioners who synthesize elements of different medical “systems.”^200^

The choices made by individuals within the global market are normally innocuous but in some cases the most affluent exercise their choice at the expense of the poorest and most vulnerable. Perhaps the most notorious example of this is the trade in organs intended for transplantation. Though this trade is distasteful to many, a number of prominent ethicists have made strong arguments in favor of it, albeit in strictly controlled circumstances.^201^ But the reality usually falls short of the ideal, for organs tend to be harvested from the desperate for substantially less than “market price.” There is also plenty of evidence to show that they are obtained under duress, especially from persons who fall victim to human traffickers.^202^ Organized on a global scale, these activities are difficult to detect and almost impossible to regulate. Questions of equity and efficiency also come into play when the global market is allowed to govern matters of public health, not least the development and distribution of vaccines. During the H1N1 influenza pandemic of 2009, stocks of vaccine were quickly exhausted as they were purchased by affluent nations. Practically none of the vaccine reached African countries, but ironically much of the unused stock from rich countries was dumped there.^203^ Without something like a global fund to purchase vaccines or, indeed, life-saving treatments for common infectious diseases, the health of many people around the world is likely to remain dependent on the good offices of charitable foundations.

## Conclusion

In this article I have made a case for the relevance of global history to historians of health, disease, and medicine. It has not been my intention to[Other P-686] be prescriptive or to devalue other approaches but merely to affirm that a global perspective can illuminate some of the central problems of our field. Widening the geographical range of our scholarship allows us to see familiar stories—like the emergence of modern “Western” medicine—in a new light, for its origins appear more diverse and exotic than traditional accounts allow. A global perspective also enables us to see connections between what at first appear to be random events, such as the growing prevalence of many diseases (human, plant, and animal; infectious and noninfectious) simultaneously in different parts of the world. Indeed, the advent of the first global market produced an epidemiological upheaval comparable to the great “exchanges” of the past. All manner of pathogens came to circulate the globe, while the environments receiving them were transformed by commercial agriculture and industrial enterprise. And yet, the world was “unified” by disease in only the most superficial sense. It soon became clear that a divergence had occurred, for the countries that gained most from global integration enjoyed improving health while conditions often worsened elsewhere. Better nutrition and sanitary infrastructure in the richer nations began to reduce mortality from infectious disease, but improvements occurred fitfully or considerably later in their colonies. It was not until the 1930s that the situation began to improve. At that time, international comparisons, rising expectations, and concerns over international competitiveness led to an escalation of state intervention and more standardized conceptions of health and illness. This transition was aided by the increasing availability of Western pharmaceuticals and services, particularly in industrial and commercial centers that had grown with the global market. These products paved the way for medical and sanitary interventions by normalizing Western medicine and the biological concepts that underpinned it.

Our present era bears some resemblance to this earlier period of integration. Once again, there is an increasing threat from certain infectious diseases, but with the exception of tuberculosis and influenza these are different from those that plagued the nineteenth century. There are other differences, too. Chronic diseases and degenerative conditions once found predominantly in the West are now ubiquitous, while health inequalities within nations—even affluent ones—are almost as striking as those which exist between them. This gradual shift reflects the emergence of a social structure that has global dimensions and of a distribution of power that is less geographically localized than in the age of empires. The response to health problems arising from global integration is also rather different. Although the organizations involved in public health and health care have always been diverse, the period from roughly 1850 to 1970 was[Other P-687] characterized by the growth of the state. Its increasing role in matters of health reflected a belief that government intervention ameliorated social inequalities and that the welfare of all classes was mutually dependent.^204^ International organizations and agreements on health revealed a similar consensus. Though spurious in many respects, this internationalism brought real improvements and helped to stabilize the global economy.^205^

Today, there is no real consensus on how to deal with the health problems arising from global integration. Whereas previous generations looked primarily to the state, its role in the provision of health care is decreasing in rich and poor countries alike. While global threats to health appear to have increased, the authority of global institutions has not. Nevertheless, “global health” is an ideal that most governments, NGOs, and charitable foundations profess. Programs of global health exist in most major universities, and the subject’s disciplinary identity is reflected in the titles of journals and monographs. All over the world, the young and idealistic are drawn to work in a field which appears to embody humanitarian ideals. But global health remains an elusive concept. It is not at all clear whether it is a noble aspiration or a new type of policy that transcends the concerns of nation-states.^206^ Both of these meanings coexist in institutions such as the WHO, which sees its role as being to promote health as a human right as well as to protect humanity from universal threats. However, the mantle of global health often disguises other motives. The term frequently dignifies the pursuit of national interests, not simply the protection of borders but foreign health interventions that are designed primarily to secure economic and political objectives.

It would be unrealistic to expect otherwise, especially in view of the competitive pressures exerted by globalization. But global health can be more than an aggregation of interests. While public health has always been part of statecraft, it has also—since its inception—reflected a shared understanding of health as a common good.^207^ For the past five centuries,[Other P-688] these ideas have been confined to the citizens and subjects of nation-states, but there are signs that our idea of the “common good” is becoming more inclusive, partly as a result of exposure to global threats.^208^ Some aspects of health policy—particularly those grounded in a narrow conception of security—have inhibited the development of this new cosmopolitanism, so the way forward, perhaps, is to conceive of security in a different way: to recognize that it is social in nature, enforced by the state—yes—but based ultimately upon trust and mutual respect.^209^ Just as public health provided the social glue that enabled nation-states to survive the turmoil of industrialization, global health may yet provide a shared identity that inspires trust and confidence between different nations and ethnicities.[Other P-689]

## Footnotes

1
HeldD. L.McGrewA. G.GoldblattD.PerratonD.Global Transformations: Politics, Economics and CultureStanfordStanford University Press1999

2
HopkinsA. G.Globalization in World HistoryLondonPimlico2002

3
O’BrienPatrickHistoriographical Traditions and Modern Imperatives for the Restoration of Global HistoryJ. Global Hist12006339

4
CooperFrederickColonialism in Question: Theory, Knowledge, HistoryBerkeleyUniversity of California Press2005

5
HodgesSarahThe Global MenaceSoc. Hist. Med2520127197282634546910.1093/shm/hkr166PMC4558944

6
PickstoneJohn V.Ways of Knowing: A New History of Science, Technology and MedicineChicagoUniversity of Chicago Press2000

7
GillsB. K.ThompsonW. R.Globalization and Global HistoryLondonRoutledge2006

8
MazlishB.IntroductionConceptualizing Global HistoryMazlishB.BuultjensR.Boulder, Colo.Westview1993126 GrafeReginaTurning Maritime History into Global History: Some Conclusions from the Impact of Globalization in Early Modern SpainResearch in Maritime History. No. 43: Maritime History as Global HistoryFusaroM.PolóniaA.St. John’s, Nfld.International Maritime Economic History Association2010249266

9
SubrahmanyamSanjayConnected Histories: Notes towards a Reconfiguration of Early Modern EurasiaMod. Asian Stud311997735762 CurthoysA.LakeM.Connected Worlds: History in Transnational PerspectiveCanberraANU Press2005

10
MazlishBruceComparing Global History to World HistoryJ. Interdiscip. Hist281998385395

11
ParkerGeoffreyThe Military Revolution: Military Innovation and the Rise of the West, 1500–1800CambridgeCambridge University Press1988 McNeillWilliam H.The Rise of the West: A History of the Human Community1963ChicagoUniversity of Chicago Press1991 HuntingtonSamuel P.The Clash of Civilizations and the Remaking of the World OrderNew YorkSimon & Schuster1996

12
BaylyC. A.The Birth of the Modern World 1780–1914OxfordBlackwell2004 McNeillJ. R.McNeillWilliam H.The Human Web: A Bird’s-Eye View of World HistoryNew YorkNorton2003 CurtinPhilip D.Cross-Cultural Trade in World HistoryNew YorkCambridge University Press1984

13
OsterhammelJürgenPetersonNeils P.Globalization: A Short HistoryPrinceton, N.J.Princeton University Press200512

14
MooreKarlLewisDavid CharlesOrigins of GlobalizationNew YorkRout-ledge2009 FlynnD. O.GiráldezA.Born Again: Globalization’s Sixteenth Century Origins (Asian/Global versus European Dynamics)Pacific Econ. Rev132008359387

15
OsterhammelPetersonGlobalizationviii

16
WallersteinImmanuelThe Modern World System3New YorkAcademic Press1974 WallersteinWorld-Systems Analysis: An IntroductionDurham, N.C.Duke University Press2004 SandersonStephen K.Civilizations and World Systems: Studying World-Historical ChangeWalnut Creek, Calif.AltaMira Press1995

17
HopkinsGlobalization in World History4773

18
WashbrookDavidIndia in the Early Modern World Economy: Modes of Production, Reproduction and ExchangeJ. Global Hist2200787111 PrakashOmEuropean Commercial Enterprise in Pre-colonial IndiaCambridgeCambridge University Press1998

19
BaylyBirth of the Modern World27

20
MarshallP.J.The Eighteenth Century in Indian History: Evolution or Revolution?New DelhiOxford University Press2003 MarshallP. J.Bengal: The British Bridgehead: Eastern India, 1740–1828CambridgeCambridge University Press1987

21
de VriesJanThe Industrious Revolution: Consumer Behavior and the Household Economy, 1650 to the PresentCambridgeCambridge University Press2008 BergMaxineLuxury and Pleasure in Eighteenth-Century BritainOxfordOxford University Press2007 CainP. J.HopkinsA. G.British Imperialism, 1688–2000LondonLongman2002

22
HeadrickDaniel R.Power over Peoples: Technology, Environments, and Western Imperialism, 1400 to the PresentPrinceton, N.J.Princeton University Press2010

23
FindlayRonaldO’RourkeKevinPower and Plenty: Trade, War, and the World Economy in the Second MillenniumPrinceton, N.J.Princeton University Press2007

24
BellDuncanVictorian Visions of Global Order: Empire and International Relations in Nineteenth-Century Political ThoughtCambridgeCambridge University Press2007

25
RobertsonRobbieThe Three Waves of Globalization: A History of a Developing Global ConsciousnessLondonZed Books2004

26
ManelaErezThe Wilsonian Moment: Self-Determination and the International Origins of Anticolonial NationalismNew YorkOxford University Press2007 KnockThomas J.To End All Wars: Woodrow Wilson and the Quest for a New World OrderPrinceton, N.J.Princeton University Press1995

27
FriedenJeffry A.Global Capitalism: Its Fall and Rise in the Twentieth CenturyNew YorkNorton2006

28
HarveyDavidThe Condition of Post-modernity: An Enquiry into the Origins of Cultural ChangeOxfordBlackwell1989 GiddensAnthonyThe Consequences of ModernityCambridgePolity1991

29
AppaduraiArjunDisjuncture and Difference in the Global Cultural EconomyTheory Cult. Soc71990295310

30
CrosbyAlfred W.The Columbian Exchange: Biological and Cultural Consequences of 1492Westport, Conn.Greenwood1972 CrosbyEcological Imperialism: The Biological Expansion of Europe, 900–1900CambridgeCambridge University Press1986 McNeillWilliam H.Plagues and PeoplesGarden City, N.Y.Anchor Press1976

31
HaysJ. N.The Burdens of Disease: Epidemics and Human Response in Western HistoryNew Brunswick, N.J.Rutgers University Press2000 HarrisonMarkDisease and the Modern World: 1500 to the Present DayCambridgePolity2004

32
RosenGeorgeWhat Is Social Medicine? A Genetic Analysis of the ConceptFrom Medical Police to Social Medicine: Essays on the History of Health CareNew YorkScience History Publications197460119

33
ArnoldDavidIntroduction: Disease, Medicine and EmpireImperial Medicine and Indigenous SocietiesArnoldD.ManchesterManchester University Press1988126

34
StannardDavid E.American Holocaust: Columbus and the Conquest of the New WorldOxfordOxford University Press1992 BrooksFrancis J.Revising the Conquest of Mexico: Smallpox, Sources, and PopulationsJ. Interdiscip. Hist2419931291528

35
CookNoble DavidBorn to Die: Disease and New World Conquest, 1492–1650CambridgeCambridge University Press1998 McCaaRobertSpanish and Nahuatl Views on Smallpox and Demographic Catastrophe in MexicoHealth and Disease in Human History: A Journal of Interdisciplinary History ReaderRotbergR.Cambridge, Mass.MIT Press2000167202

36
KunitzStephen J.Disease and Social Diversity: The European Impact on the Health of Non-EuropeansNew YorkOxford University Press1994

37
CrosbyA. W.Hawaiian Depopulation as a Model for the Amerindian ExperienceEpidemics and Ideas: Essays on the Historical Perception of PestilenceRangerT.SlackP.CambridgeCambridge University Press1992175201

38
LadurieEmmanuel Le RoyA Concept: The Unification of the Globe by DiseaseMind and Method of the HistorianReynoldsSiânReynoldsBenBrightonHarvester19812883

39
McNeillJ. R.Mosquito Empires: Ecology and War in the Greater Caribbean, 1620–1914CambridgeCambridge University Press2010

40
de VriesJanThe Limits of Globalization in the Early Modern WorldEcon. Hist. Rev632010710733

41
BenedictowOle J.The Black Death 1346–1353: The Complete HistoryWoodbridgeBoydell Press2004

42
ArrizabalagaJonHendersonJohnFrenchRogerThe Great Pox: The French Disease in Renaissance EuropeNew Haven, Conn.Yale University Press1997

43
BosKirsten I.SchuenemannVerena J.GoldingG. BrianA Draft Genome of *Yersinia pestis* from Victims of the Black DeathNature47820115065102199362610.1038/nature10549PMC3690193 MorelliGiovanna*Yersinia Pestis* Genome Sequencing Identifies Patterns of Global Phylogenetic DiversityNature Genet422010114011432103757110.1038/ng.705PMC2999892

44
CampbellBruce M. S.Physical Shocks, Biological Hazards, and Human Impacts: The Crisis of the Fourteenth Century RevisitedLe interazioni fra economia e ambiente biologico nell’Europe Preindustriale Secc. XIII–XVIIICavaciocchiS.PratoF. Datini20101332 SussmanGeorge D.Was the Black Death in India and China?Bull. Hist. Med8520113193552208079510.1353/bhm.2011.0054 LeungAngela Ki CheDiseases of the Premodern Period in ChinaThe Cambridge World History of Human DiseaseKipleK. F.CambridgeCambridge University Press1993354362 BozhongLiWas There a ‘Fourteenth-Century Turning Point’? Population, Land, Technology and Farm ManagementThe Song-Yuan-Ming Transition in Chinese HistorySmithP. J.von GlahnR.Cambridge, Mass.Harvard University Press2003134175

45
A Voyage Round the World by Dr. John Francis Gernelli Careri, Containing the Most Remarkable Things He Saw in IndostanIndia in the Seventeenth Century2GuhaJ. P.New DelhiAssociated Publishing House1976203 Tuzuk-i-JahangiriAhmudSyudAligharhPrivate Press1864209210219220

46
ArnoldDavidThe Indian Ocean as a Disease Zone, 1500–1950South Asia141991122

47
PrinsGwynBut What Was the Disease? The Present State of Health and Healing in African StudiesPast Pres124198915917910.1093/past/124.1.15911620418

48
RosenWilliamJustinian’s Flea: Plague, Empire and the Birth of EuropeLondonViking2007 LittleLester K.Plague and the End of Antiquity: The Pandemic of 541–750CambridgeCambridge University Press2007 BorschStuart J.The Black Death in Egypt and England: A Comparative StudyAustinUniversity of Texas Press2005 DolsMichael W.The Black Death in the Middle EastPrinceton, N.J.Princeton University Press1977

49
WhiteSamThe Climate of Rebellion in the Early Modern Ottoman EmpireCambridgeCambridge University Press2011 WhiteRethinking Disease in Ottoman HistoryInt. J. Middle East Stud422010549567 VarlikNükhetPlague and Empire in the Early Modern Mediterranean World: The Ottoman Experience, 1347–1600New York and CambridgeCambridge University Press2015

50 Works on this subject are too numerous to mention, but for an indicative and influential sample see the essays in Ranger and Slack, *Epidemics and Ideas* (n. 37).

51
LadurieA Concept30

52
GeggusDavidSlavery, War, and Revolution: The British Occupation of Saint Domingue, 1793–1798OxfordClarendon1982 EstesJ. WorthSmithB. G.A Melancholy Scene of Devastation: The Public Response to the 1793 Philadelphia Yellow Fever EpidemicPhiladelphiaCollege of Physicians of Philadelphia1997 TaylorP. Sean‘We Live in the Midst of Death’: Yellow Fever, Moral Economy and Public Health in Philadelphia, 1793–1805Ph.D. diss.Northern Illinois University2001 PowellJ. H.Bring Out Your Dead: The Great Plague of Yellow Fever in Philadelphia in 1793PhiladelphiaUniversity of Pennsylvania Press1949

53
ArtiagaMercedes PascualFam, malatia I mort: Alacant I la fibre groga de l’any 1804Simat de la ValldignaLa Xara2000 GarciaM. Dolores GasparLa epidemia de fiebre amarilla que asoló Barcelona en 1821, a través del contenido del manuscrito 156 de la Biblioteca Universitaria de BarcelonaGimbernat1819926572 CabalM.Medidas adoptadas por la Junta de Sanidad del Principado ante la posible invasión peninsular de la fiebre amarilla existente en Cádiz y Real Isla de León en el siglo XVIII al XIXBoletín del Instituto de Estudios Asturianos421988409428

54
ColemanWilliamYellow Fever in the North: The Methods of Early EpidemiologyMadisonUniversity of Wisconsin Press1987

55
HamlinChristopherCholera: The BiographyOxfordOxford University Press2009 MukharjiProjit BihariThe ‘Cholera Cloud’ in the Nineteenth-Century ‘British World’: History of an Object-Without-an-EssenceBull. Hist. Med8620123033322324190810.1353/bhm.2012.0050 EchenbergMyronAfrica in the Time of Cholera: A History of Pandemics from 1817 to the PresentCambridgeCambridge University Press2011 BaldwinPeterContagion and the State in Europe, 1830–1930CambridgeCambridge University Press1999

56
JonesDavidRationalizing Epidemics: Meanings and Uses of American Indian Mortality Since 1600Cambridge, Mass.Harvard University Press2004 CampbellJudyInvisible Invaders: Smallpox and Other Diseases in Aboriginal Australia 1780–1880CarltonMelbourne University Press2002

57
EchenbergMyronPlague Ports: The Global Urban Impact of Bubonic Plague, 1894–1901New YorkNew York University Press2007

58
HonigsbaumMarkThe Great Dread: Cultural and Psychological Impacts and Responses to the ‘Russian’ Influenza in the United Kingdom, 1889–1893Soc. Hist. Med232010299319 PhillipsHowardKillingrayDavidThe Spanish Influenza Pandemic of 1918–19: New PerspectivesLondonRoutledge2003

59
BrownKarenMad Dogs and Meerkats: A History of Resurgent Rabies in Southern AfricaAthensOhio University Press2011 MishraSaurabhBeasts, Murrains and the *Raj*: Reassessing Colonial Medicine in India from the Veterinary Perspective, 1860–1900Bull. Hist. Med8520115876192250643410.1353/bhm.2011.0089PMC3477853 JonesSusan D.Death in a Small Package: A Short History of AnthraxBaltimoreJohns Hopkins University Press2010 BrownKarenGilfoyleDanielHealing the Herds: Disease, Livestock Economies, and the Globalization of Veterinary MedicineAthensOhio University Press2010 BeinartWilliamHughesLotteEnvironment and EmpireOxfordOxford University Press2007 WoodsAbigailA Manufactured Plague: The History of Foot-and-Mouth Disease in BritainLondonEarthscan2004 PhoofoloPuleFace to Face with Famine: The BaSotho and the Rinderpest, 1897–1899J. South African Stud292003503527 CranefieldPaulScience and Empire: East Coast Fever in Rhodesia and the TransvaalCambridgeCambridge University Press1991 OfcanskyThomas P.The 1889–97 Rinderpest Epidemic and the Rise of British and German Colonialism in Eastern and Southern AfricaJ. African Stud81981313811614677 MachadoManuel A.Aftosa: A Historical Survey of Foot-and-Mouth Disease and Inter-American RelationsAlbanyState University of New York Press1969

60
DonnellyJames S.The Great Irish Potato FamineStroudSutton2001 DixAndreasPhylloxeraEncyclopedia of World Environmental HistoryKrechS.McNeillJ. R.MerchantC.LondonRoutledge20041002 ClarkJ. F. M.Bugs and the VictoriansNew Haven, Conn.Yale University Press2009

61
HarrisonMarkContagion: How Commerce Has Spread DiseaseNew Haven, Conn.Yale University Press2012216218

62
HarrisonMarkDisease and World History from 1750The Cambridge World History Volume 7: Production, Destruction and Connection, 1750–Present, Part 1: Structures, Spaces, and Boundary MakingMcNeillJohnPomeranzKennethNew YorkCambridge University Press2015237257 MishraSaurabhPilgrimage, Politics, and Pestilence: The Haj from the Indian Subcontinent, 1860–1920New DelhiOxford University Press2011 Smallman-RaynorM. R.CliffA. D.War Epidemics: An Historical Geography of Infectious Diseases in Military Conflict and Civil Strife, 1850–2000OxfordOxford University Press2004 CurtinPhilip D.Migration and Mortality in Africa and the Atlantic World, 1700–1900AldershotAshgate2001 MarksL.WorboysM.Migrants, Minorities and HealthLondonRoutledge1997

63
PolanyiKarlThe Great Transformation: The Political and Economic Origins of Our Time1944BostonBeacon2001

64
HarleyC. K.The Integration of the World Economy, 1850–1914CheltenhamE. Elgar1996 LathamA. J. H.The International Economy and the Underdeveloped World, 1865–1914LondonCroom Helm1978

65
BairochPaulGlobalization Myths and Realities: One Century of External Trade and Foreign InvestmentStates Against Markets: The Limits of GlobalizationBoyerR.DracheD.LondonRoutledge1996128142 WilliamsonJeffrey G.Globalization, Convergence, and HistoryJ. Econ. Hist561996277306 WilliamsonThe Evolution of Global Labor Markets since 1830: Background, Evidence and HypothesesExplor. Econ. Hist321995141196 TaylorAlan M.WilliamsonJeffrey G.Convergence in the Age of Mass MigrationEur. Rev. Econ. Hist119972763 O’RourkeKevinWilliamsonJeffery G.Late-Nineteenth-Century Anglo-American Factor-Price Convergence: Were Heckscher and Ohlin Right?J. Econ. Hist541994892916

66
RiviereW. E.Labour Shortage in the British West Indies after EmancipationJ. Caribbean Hist41972130 LaiWalton LookIndentured Labor, Caribbean Sugar: Chinese and Indian Migrants to the British West Indies, 1838–1918BaltimoreJohns Hopkins University Press1993 NorthrupDavidIndentured Labor in the Age of Imperialism, 1834–1922CambridgeCambridge University Press1995 KaleMadhaviFragments of Empire: Capital, Slavery, and Indian Indentured Labor Migration in the British CaribbeanPhiladelphiaUniversity of Pennsylvania Press1998

67
ShlomowitzRalphMcDonaldJohnMortality of Indian Labour on Ocean Voyages, 1843–1917Stud. Hist619903565 ShlomowitzRalphMortality and Migration in the Modern WorldAldershotVariorum1996 BrownLaurenceMahaseRadicaMedical Encounters on the *Kala Pani*: Regulation and Resistance in the Passages of Indentured Indian Migrants, 1834–1900Health and Medicine at Sea, 1700–1900HaycockD. B.ArcherS.WoodbridgeBoydell Press2009195212

68
WebbJames L. A.Jr.Humanity’s Burden: A Global History of MalariaNew YorkCambridge University Press2009115116

69
BhattacharyaNandiniContagion and Enclaves: Tropical Medicine in Colonial IndiaLiverpoolLiverpool University Press2012 DuttaAchintya Kumar*Kala-Azar* in Assam: British Medical Intervention and People’s ResponseMaladies, Preventives and Curatives: Debates in Public Health in IndiaBagchiA. K.SomanK.New DelhiTulika Books20051531

70
KerrIan J.Building the Railways of the Raj, 1850–1900New DelhiOxford University Press1997159161 ShlomowitzRalphBrennanLanceMortality and Migrant Labour en route to Assam, 1863–1924Indian Econ. Soc. Hist. Rev271990313330

71
PalmerStevenLaunching Global Health: The Caribbean Odyssey of the Rockefeller FoundationAnn ArborUniversity of Michigan Press2010 FarleyJohnTo Cast Out Disease: A History of the International Health Division of the Rockefeller Foundation, 1913–1951New YorkOxford University Press2004 CuetoMarcosMissionaries of Science: The Rockefeller Foundation and Latin AmericaBloomingtonUniversity of Indiana Press1994 EttlingJohnThe Germ of Laziness: Rockefeller Philanthropy and Public Health in the New SouthCambridge, Mass.Harvard University Press1981

72
HewaSomaColonialism, Tropical Disease and Imperial Medicine: Rockefeller Philanthropy in Sri LankaLanham, Md.University Press of America1995 HewaThe Hookworm Epidemic on the Plantations in Colonial Sri LankaMed. Hist3819947390814560910.1017/s0025727300056052PMC1036811

73
PackardRandallThe Making of a Tropical Disease: A Short History of MalariaBaltimoreJohns Hopkins University Press2007 RichellJudith L.Disease and Demography in Colonial BurmaSingaporeNUS Press2006 BellHeatherFrontiers of Medicine in the Anglo-Egyptian Sudan 1899–1940OxfordClarendon1999 WattsSheldonBritish Development Policies and Malaria in India 1897–*c*.1929Past Pres165199914118110.1093/past/165.1.14122043526 StoneIanCanal Irrigation in British India: Perspectives on Technological Change in a Peasant EconomyCambridgeCambridge University Press1984144158

74 See Packard, *Making of a Tropical Disease* (n. 73); Webb, *Humanity’s Burden* (n. 68).

75
LaventureS.MouchetJ.BlanchyS.MarramaL.Le riz source de vie et de mort sur les plateaux de MadagascarCahiers d’études et des recherches francophones/Santé6199679868705134

76
WhitcombeElizabethThe Environmental Costs of Irrigation in British India: Water-logging, Salinity and MalariaNature, Culture, Imperialism: Essays on the Environmental History of South AsiaArnoldD.GuhaR.DelhiOxford University Press1996237259 WakimuraKoheiFamines, Epidemics and Mortality in Northern IndiaLocal Agrarian Societies in Colonial India: Japanese PerspectivesRobbP.SugiharaK.YanagisawaH.LondonCurzon Press1996280310 HarrisonMarkDisease and the Dilemmas of Development: A Malaria Strategy for Bombay, 1902–428th Hasi Majumdar Oration on History and Philosophy of Medicine and ScienceUniversity of Calcutta3222011142

77
AckerknechtErwin H.Malaria in the Upper Mississippi Valley, 1760–1900BaltimoreJohns Hopkins University Press1945

78
SnowdenFrank M.The Conquest of Malaria: Italy, 1900–1962New Haven, Conn.Yale University Press20062526

79
BayAlexander R.Beriberi in Modern Japan: The Making of a National DiseaseRochester, N.Y.Rochester University Press2012 ArnoldDavidBritish India and the ‘Beriberi Problem’ 1798–1942Med. Hist5420102953142059288210.1017/s0025727300004622PMC2889456 CarpenterKenneth J.Beriberi, White Rice, and Vitamin B: A Disease, a Cause, and a CureBerkeleyUniversity of California Press2000 MandersonLenoreSickness and the State: Health and Illness in Colonial Malaya, 1870–1940CambridgeCambridge University Press19968992

80
BryderLindaTuberculosis, Silicosis, and the Slate Industry in North Wales 1927–1939The Social History of Occupational HealthWeindlingP.LondonCroom Helm1985108126

81
HamlinChristopherPublic Health and Social Justice in the Age of Chadwick: Britain, 1800–1854CambridgeCambridge University Press1998174

82
McIvorArthurGerms at Work: Establishing Tuberculosis as an Occupational Disease in Britain, *c*.1900–1951Soc. Hist. Med252012812829

83
PackardRandallWhite Plague, Black Labor: Tuberculosis and the Political Economy of Health and Disease in South AfricaBerkeleyUniversity of California Press1989

84
MarksWorboysMigrants, Minorities and Health93124

85
ChoenniChan E. S.From Bharat to Sri Ram Desh: The Emigration of Indian Indentured Labourers to SurinameThe South Asian Diaspora: Transnational Networks and Changing IdentitiesRaiR.ReevesP.LondonRoutledge2009108123 FairchildAmyScience at the Borders: Immigrant Medical Inspection and the Shaping of the Modern Industrial Labor ForceBaltimoreJohns Hopkins University Press2003

86
HarrisonMarkThe Medical War: British Military Medicine in the First World WarOxfordOxford University Press2010 WeindlingPaulEpidemics and Genocide in Eastern Europe, 1890–1945OxfordOxford University Press2000

87
HuberValeskaThe Unification of the Globe by Disease? The International Sanitary Conferences on Cholera, 1851–1894Hist. J492006453476 HarrisonMarkDisease, Diplomacy and International Commerce: The Origins of International Sanitary Regulation in the Nineteenth CenturyJ. Global Hist12006197217 HarrisonQuarantine, Pilgrimage and Colonial Trade: India 1866–1900Indian Econ. Soc. Hist. Rev291992117144

88
HarrisonContagion122138196210 OsterhammelJürgenDie Verwandlung der Welt: Eine Geschichte des 19. JarhhundertsMunichC.H. Beck2009293294

89
ChiffoleauSylviaGenèse de la santé publique internationale: De la peste d’Orient à l’OMSRennesPresses Universitaire de Rennes2012 PailletteCélineDe l’Organisation d’hygiène de la SDN à l’OMS: mondialisation et regionalism européen dans le domaine de la santé, 1919–1954Bulletin de l’Institut Pierre Renouvin322010238253 CuetoMarcosThe Value of Health: A History of the Pan American Health OrganizationWashington, D.C.Pan American Health Organization2007 RasmussenAnneL’hygiène en congrès (1852–1912): circulation et configurations internationalesLes hygiénistes, enjeux, modèles et pratiquesBourdelaisP.ParisBelin2001213239

90
HarleyC. KnickSteers Afloat: The North Atlantic Meat Trade, Liner Predominance, and Freight Rates, 1870–1913J. Econ. Hist68200810281058 HoySuellenNugentWalterPublic Health or Protectionism? The German-American Pork War, 1880–1891Bull. Hist. Med6319891982242667661 GignilliatJohn L.Pigs, Politics and Protection: The European Boycott of American Pork, 1879–1891Agric. Hist351961312

91
MahaseRadicaHealth and Medical Care of Indian Indentured Labourers in Trinidad: Imperialist Contradictions, 1870–1917Global South: *Sephis*520091419

92
BasuShuboThe Emergence of the Mill Towns in Bengal, 1880–1920: Migration Patterns and Survival Strategies of Industrial WorkersCalcutta Hist. J18199697133 JonesMargaretThe Indian Immigrant Worker and the Development of Hospital Provision in Nineteenth-Century CeylonFrom Western Medicine to Global Medicine: The Hospital Beyond the WestHarrisonM.JonesM.SweetH.New DelhiOrient Black-Swan20093366

93 Packard, *White Plague, Black Labor* (n. 83).

94
CrossleyPamelaWhat Is Global History?CambridgePolity2007

95
WorboysMichaelBefore McKeown: Explaining the Decline of Tuberculosis in Britain, 1880–1930Tuberculosis Then and NowCondrauF.WorboysM.MontrealMcGill-Queens University Press2010148170 FloudRoderickFogelRobert W.HarrisBernardHongSok ChulThe Changing Body: Health, Nutrition, and Human Development in the Western World since 1700CambridgeCambridge University Press201110.1016/j.jeca.2011.02.002PMC458313626413098

96
FarmerPaulInfections and Inequalities: The Modern PlaguesBerkeleyUniversity of California Press1999

97
CoxLloydThe Antipodean Social Laboratory, Labour and the Transformation of the Welfare StateJ. Sociol422006107124

98 See, e.g., Packard, *Making of a Tropical Disease* (n. 73).

99
AndersonWarwickPostcolonial Histories of MedicineLocating Medical History: The Stories and Their MeaningsHuismanF.WarnerJ. H.BaltimoreJohns Hopkins University Press2004285306 ChakrabartyDipeshProvincializing Europe: Postcolonial Thought and Historical DifferencePrinceton, N.J.Princeton University Press2000 GhoshD.KennedyD.Decentring Empire: Britain, India and the Transcolonial WorldHyderabadOrient Longman2006

100
GarrisonFielding H.An Introduction to the History of MedicinePhiladelphiaSaunders1917 AckerknechtErwin H.A Short History of MedicineBaltimoreJohns Hopkins University Press1968

101
SigeristHenryA History of Medicine: Volume II: Early Greek, Hindu, and Persian MedicineNew YorkOxford University Press1987

102
PorterRoyThe Greatest Benefit to Mankind: A Medical History of Humanity from Antiquity to the PresentLondonHarperCollins1997

103
Shefer-MossensohnMiriOttoman Medicine: Healing and Medical Institutions, 1500–1700AlbanyState University of New York Press2009 AlaviSeemaIslam and Healing: Loss and Recovery of an Indo-Muslim Medical Tradition, 1600–1900BasingstokePalgrave Macmillan2008

104
KeelOthmarL’Avènement de la Médicine Clinique Moderne en Europe: 1750–1815: Politiques, Institutions et SavoirsMontrealPresses de l’Université de Montréal2001 BergeA. LaHannawayC.Constructing Paris MedicineAmsterdamRodopi1998 BynumW. F.Science and the Practice of Medicine in the Nineteenth CenturyCambridgeCambridge University Press1994 FoucaultMichelThe Birth of the Clinic: An Archaeology of Medical PerceptionSheridanA. M.LondonRoutledge1997 AckerknechtErwin H.Medicine at the Paris Hospital, 1794–1848BaltimoreJohns Hopkins University Press1967

105
ChakrabartiPratikMaterials and Medicine: Trade, Conquest and Therapeutics in the Eighteenth CenturyManchesterManchester University Press2010 CookHarold J.Matters of Exchange: Commerce, Medicine, and Science in the Dutch Golden AgeNew Haven, Conn.Yale University Press2007 HarrisSteven J.Long-Distance Corporations, Big Sciences, and the Geography of KnowledgeConfigurations61998269304

106
CookHarold J.WalkerTimothy D.Circulation of Medicine in the Early Modern Atlantic WorldSoc. Hist. Med262013337351 SchiebingerLondaPlants and Empire: Colonial Bioprospecting in the Atlantic WorldCambridge, Mass.Harvard University Press2004 SchiebingerL.SwanC.Colonial Botany: Science, Commerce, and Politics in the Early Modern WorldPhiladelphiaUniversity of Pennsylvania Press2005 GroveRichardIndigenous Knowledge and the Significance of South-West India for Portuguese and Dutch Constructions of Tropical NatureMod. Asian Stud301996121143 PearsonM. N.The Thin End of the Wedge: Medical Relativities as a Paradigm of Early Modern Indian-European RelationsMod. Asian Stud291996141170 HarrisonMarkMedicine and Orientalism: Perspective on Europe’s Encounter with Indian Medical SystemsHealth, Medicine and Empire: Perspectives on Colonial IndiaPatiB.HarrisonM.HyderabadOrient Longman20013787 PolsHansEuropean Physicians and Botanists, Indigenous Herbal Medicine in the Dutch East Indies, and Colonial Networks of MediationEast Asian Sci. Technol. Soc32009173208

107
HarrisonMarkMedicine in an Age of Commerce and Empire: Britain and Its Tropical Colonies, 1660–1830OxfordOxford University Press2010

108
HarrisonMarkThe Calcutta Botanic Garden and the Wider World, 1817–46Science and Modern India: An Institutional History, c.1784–1947GuptaU. DasDelhiPearson Longman2011235253

109
AdasMichaelMachines as the Measure of Men: Science, Technology, and Ideologies of Western DominanceIthaca, N.Y.Cornell University Press1989

110
HardimanDavidHealing Bodies, Saving Souls: Medical Missions in Asia and AfricaAmsterdamRodopi200617132180

111 See Harrison, Jones, and Sweet, *From Western Medicine to Global Medicine* (n. 92).

112
MonnaisL.CookH.J.Global Movements, Local Concerns: Medicine and Health in Southeast AsiaSingaporeNUS Press2012 MonnaisLaurence‘Modern Medicine’ in French Colonial Vietnam: From the Importation of a Model to Its NativisationThe Development of Modern Medicine in Non-Western CountriesEbrahimnejadH.LondonRoutledge2009127159 MukharjiProjitNationalizing the Body: The Medical Market, Print and Daktari MedicineLondonAnthem2009 PrakashGyanAnother Reason: Science and the Imagination of Modern IndiaPrinceton, N.J.Princeton University Press1999

113
PeardJulyan G.Race, Place and Medicine: The Idea of the Tropics in Nineteenth-Century Brazilian MedicineDurham, N.C.Duke University Press1999 HaynesDouglas M.Imperial Medicine: Patrick Manson and the Conquest of Tropical DiseasePhiladelphiaUniversity of Pennsylvania Press2001 AndersonWarwickColonial Pathologies: American Tropical Medicine, Race, and Hygiene in the PhilippinesDurham, N.C.Duke University Press2006

114
KellerRichard C.Colonial Madness: Psychiatry in French North AfricaChicagoUniversity of Chicago Press2007

115
NeillDeborah J.Networks in Tropical Medicine: Internationalism, Colonialism, and the Rise of a Medical Speciality, 1890–1930StanfordStanford University Press2012

116
BhattacharyaSanjoyHarrisonMarkWorboysMichaelFractured States: Smallpox, Public Health and Vaccination Policy in British India, 1800–1947New DelhiOrient Longman2005

117
KumarAnilMedicine and the Raj: British Medical Policy in India, 1835–1911New DelhiSage19981722

118
BalaPoonamBiomedicine as a Contested Site: Some Revelations in Imperial ContextsLanham, Md.Lexington Books2009 SutphenM.AndrewsB.Medicine and Colonial IdentityLondonRoutledge2003 CunninghamA.AndrewsB.Western Medicine as Contested KnowledgeManchesterManchester University Press1997

119
BowersJohn Z.When the Twain Meet: The Rise of Western Medicine in JapanBaltimoreJohns Hopkins University Press1980 LoMing-CheugDoctors within Borders: Profession, Ethnicity, and Modernity in Colonial TaiwanBerkeleyUniversity of California Press2002 TakahashiAyaThe Development of the Japanese Nursing Profession: Adopting and Adapting Western InfluencesLondonRoutledge Curzon2004 LowM.Building a Modern Japan: Science, Technology, and Medicine in the Meiji Era and BeyondBasingstokePalgrave Macmillan2005

120 Ebrahimnejad, *Development of Modern Medicine* (n. 112).

121
HarrisonMarkPublic Health in British India: Anglo-Indian Preventive Medicine, 1859–1914CambridgeCambridge University Press199417

122
SharmaMadhuriIndigenous and Western Medicine in Colonial IndiaDelhiFoundation Books2011 SivaramakrishnanKavitaOld Potions, New Bottles: Recasting Indigenous Medicine in Colonial Punjab, 1850–1945HyderabadOrient Longman2006 AttewellGuyRefiguring Unani Tibb: Plural Healing in Late Colonial IndiaHyderabadOrient Longman2007

123
Zweiniger-BargielowskaInaManaging the Body: Beauty, Health and Fitness in Britain, 1880–1939OxfordOxford University Press2010 BeierLucinda McCrayFor Their Own Good: The Transformation of English Working-Class Health Culture, 1880–1970ColumbusOhio State University Press2008 HauMichaelThe Cult of Health and Beauty in Germany: A Social History, 1890–1930ChicagoUniversity of Chicago Press2003 BergdoltKlausWellbeing: A Cultural History of Healthy LivingCambridgePolity1999 WhortonJames C.Crusaders for Fitness: The History of American Health ReformersPrinceton, N.J.Princeton University Press1982

124
PorterDorothyHealth Citizenship: Essays in Social Medicine and Biomedical PoliticsBerkeleyUniversity of California Press2011 PorterHealth, Civilization and the State: A History of Public Health from Ancient to Modern TimesLondonRoutledge199910.1136/bmj.318.7190.1083PMC111547710205130 ArmstrongDavidPolitical Anatomy of the Body: Medical Knowledge in Britain in the Twentieth CenturyCambridgeCambridge University Press1983

125 Attewell, *Refiguring Unani Tibb* (n. 122); Sharma, *Indigenous and Western Medicine* (n. 122).

126
LewisBrian“So Clean”: Lord Leverhulme, Soap and CivilizationManchesterManchester University Press2008 BurkeTimothyLifebuoy Men, Lux Women: Commodification, Consumption, and Cleanliness in Modern ZimbabweDurham, N.C.Duke University Press1996

127
PeckhamRobertPomfretDavid M.Imperial Contagions: Medicine, Hygiene, and Cultures of Planning in AsiaHong KongHong Kong University Press2013 RogaskiRuthHygienic Modernity: Meanings of Health and Disease in Treaty-Port ChinaBerkeleyUniversity of California Press2004 MichaelsPaula A.Curative Powers: Medicine and Empire in Stalin’s Central AsiaPittsburghUniversity of Pittsburgh Press2003 HornDavid G.Social Bodies: Science, Reproduction and Italian ModernityPrinceton, N.J.Princeton University Press1995 WeindlingPaulHealth, Race and German Politics between National Unification and Nazism, 1870–1945CambridgeCambridge University Press1989 JonesGretaSocial Hygiene in Twentieth Century BritainLondonCoom Helm1986

128
BryderLinda‘Babies of the Empire’: The Evolution of Infant Welfare Services in New Zealand and Britain in the First Half of the Twentieth CenturyThe Practice of Reform in Health Medicine and Science, 1500–2000PellingM.MandelbroteS.AldershotAshgate2005247262 KlausenSusanne M.Race, Maternity, and the Politics of Birth Control in South Africa, 1910–39LondonPalgrave Macmillan2004 DavinAnnaImperialism and MotherhoodHistory Workshop Journal5197896510.1093/hwj/5.1.911610463

129
PickstoneJohnProduction, Community and Consumption: The Political Economy of Twentieth-Century Medicine119 SturdySteveThe Industrial Body217234 Companion to Medicine in the Twentieth CenturyCooterR.PickstoneJ.LondonHarwood2000 LuptonDeborahThe Imperative of Health: Public Health and the Regulated BodyLondonSage1995 RabinbachAnsonThe Human Motor: Energy, Fatigue and the Origins of ModernityBerkeleyUniversity of California Press1992 HarrisonMarkMedicine and Victory: British Military Medicine in the Second World WarOxfordOxford University Press2004 CooterR.HarrisonM.SturdyS.Medicine and Modern WarfareAmsterdamRodopi1999 CooterR.HarrisonM.SturdyS.War, Medicine and ModernityStroudSutton1998

130
HodgesSarahReproductive Health in India: History, Politics, ControversiesNew DelhiOrient Longman2006 ArnoldDavid‘An Ancient Race Outworn’: Malaria and Race in Colonial India, 1860–1936Race, Science and Medicine, 1700–1960ErnstW.HarrisB.LondonRoutledge1999123143 DikötterFrankThe Discourse of Race in Modern ChinaStanfordStanford University Press1994

131 See Low, *Building a Modern Japan* (n. 119).

132
TomesNancyThe Gospel of Germs: Men, Women, and the Microbe in American LifeCambridge, Mass.Harvard University Press199911623768

133
ThomsonMathewPsychological Subjects: Identity, Culture, and Health in Twentieth-Century BritainOxfordOxford University Press2006 HarringtonAnneReenchanted Science: Holism in German Culture from Wilhelm II to HitlerPrinceton, N.J.Princeton University Press199610.1177/00732753980360040411620555

134
WrenchG. T.The Wheel of HealthLondonC. W. Daniel1938 StuartTristramThe Bloodless Revolution: Radical Vegetarians and the Discovery of IndiaLondonHarperPress2008 HowardLouise E.Sir Albert Howard in IndiaLondonFaber & Faber1953 LockeretzWilliamOrganic Farming: An International HistoryOxfordCAB International2007

135
JonesMargaretPublic Health in Jamaica, 1850–1940: Neglect, Philanthropy and DevelopmentKingstonUniversity of the West Indies Press2013 BuLipingStapletonDarwinYipKa-cheScience, Public Health and the State in Modern AsiaLondonRoutledge2012 BhattacharyaSanjoyGlobal and Local Histories of Medicine: Interpretative Challenges and Future PossibilitiesThe Oxford Handbook of the History of MedicineJacksonM.OxfordOxford University Press2011135149 BirnAnne-EmanuelleMarriage of Convenience: Rockefeller International Health and Revolutionary MexicoRochester, N.Y.Rochester University Press2006

136
AmrithSunilDecolonizing International Health: India and Southeast Asia, 1930–65BasingstokePalgrave2006

137
BorowyIrisComing to Terms with World Health: The League of Nations Health Organisation 1921–1946BerlinPeter Lang Verlag2009 BashfordAlisonGlobal Biopolitics and the History of World HealthHist. Hum. Sci1920066788 WeindlingPaulInternational Health Organisations and Movements, 1918–1939CambridgeCambridge University Press1995

138
SigeristHenry E.Civilization and DiseaseChicagoUniversity of Chicago Press194359

139
FarleyJohnBrock Chisholm, the World Health Organization & the Cold WarVancouverUBC Press2008 BhattacharyaSanjoyExpunging Variola: The Control and Eradication of Smallpox in India, 1947–1977HyderabadOrient Longman2006

140
StepanNancy LeysEradication: Ridding the World of Diseases Forever?Ithaca, N.Y.Cornell University Press2011 CuetoMarcosCold War, Deadly Fevers: Malaria Eradication in Mexico, 1955–1975BaltimoreJohns Hopkins University Press2007 SiddiqiJavedWorld Health and World Politics: The World Health Organization and the U.N. SystemLondonHurst1995

141
DavisD.SiuH.SARS: Reception and Interpretations in Three Chinese CitiesLondonRoutledge2007 AbrahamThomasTwenty-First Century Plague: The Story of SARSBaltimoreJohns Hopkins University Press2004 BaldwinPeterDisease and Democracy: The Industrialized World Faces AIDSBerkeleyUniversity of California Press2005 FeeE.FoxD. M.AIDS: The Making of a Chronic DiseaseBerkeleyUniversity of California Press1992

142
BrandtAllanHow AIDS Invented Global HealthNew Engl. J. Med3682013214924522373854210.1056/NEJMp1305297

143
Giles-VernickTamaraWebbJames L. A.Jr.IntroductionGlobal Health in Africa: Historical Perspectives on Disease ControlGiles-VernickT.WebbJ. L. A.Jr.AthensOhio University Press201312134

144
PrinsGwynAIDS and Global SecurityInt. Aff802004931952

145
PetersonSusanEpidemic Infectious Diseases and National SecuritySecurity Stud1220024381

146
The Global Health Strategy of the U.S. Department of Health and Human Serviceshttp//www.globalhealth.govU.S. State Department Global Health Initiativehttp:///www.ghi.govFebruary 17, 2013

147
ChanMargaretDrReturn to Alma-AtaLancet37220088658661879029210.1016/S0140-6736(08)61372-0 FarmerPaulPrefaceReimagining Global Health: An IntroductionFarmerP.KimJ. Y.KleinmanA.BasilicoM.BerkeleyUniversity of California Press2013xiii

148
KingNicholas B.Security, Disease, Commerce: Ideologies of Postcolonial Global HealthSoc. Stud. Sci322002763789 GarrettLaurieThe Coming Plague: Newly Emerging Diseases in a World Out of BalanceLondonPenguin1995 PrestonRichardThe Hot Zone: A Terrifying True StoryNew YorkRandom House1994 Emerging Infections: Microbial Threats to Health in the United StatesWashington, D.C.National Academy Press199225121245

149
Yen-FenTsengChia-LingWuGoverning Germs from Outside and Within Borders: Controlling 2003 SARS Risks in TaiwanHealth and Hygiene in Chinese East Asia: Policies and Publics in the Long Twentieth CenturyLeungA. K. C.FurthC.Durham, N.C.Duke University Press2010255272 AliS. HarrisKeilR.Networked Disease: Emerging Infections in the Global CityOxfordWiley-Blackwell2008 BashfordAlisonMedicine at the Border: Disease, Globalization and Security, 1850 to the PresentBasingstokePalgrave Macmillan2006 BrookesTimBehind the Mask: How the World Survived SARS, the First Epidemics of the Twenty-First CenturyWashington, D.C.American Public Health Association2005 LohC.At the Epicenter: Hong Kong and the SARS OutbreakHong KongHong Kong University Press2004

150
GratzerDavidSARS 101Nat. Rev5192003 FidlerDavid P.Germs, Governance, and Global Public Health in the Wake of SARSJ. Clin. Invest11320047998041506730910.1172/JCI21328PMC362129

151 Bashford, *Medicine at the Border* (n. 149).

152
DemickBarbaraTaiwan Takes No Chances on SARSLos Angeles Times5152003http://articles.latimes.com/2003/may/15/world/fg-taisars15September 15, 2013

153
ChangIrisFear of SARS, Fear of StrangersNew York Times5212003http://www.nytimes.com/2003/05/21/opinion/21CHAN.htmlAugust 25, 2015

154
RosenbergCharles E.What Is an Epidemic? AIDS in Historical PerspectiveDaedlus118198911711612464

155
HonigsbaumMark‘The Great Dread’: Influenza in the United Kingdom in Peace and War, 1889–1919Ph.D. diss.Queen Mary, University of London2011

156
HarrisonContagion237246

157
BeckUlrichWorld at RiskCambridgePolity2009175176

158
KirktonJohn J.RoudevNikolaiSunderlandLauraMaking G8 Leaders Deliver: An Analysis of Compliance and Health Commitments, 1996–2006Bull. World Health Organization85200719219910.2471/BLT.06.039917PMC263623317486210 FidlerDavid P.GostinLawrence O.The New International Health Regulations: An Historic Development for International Law and Public HealthJ. Law Med. Ethics200685941648998710.1111/j.1748-720X.2006.00011.x CooperA. F.KirtonJ. J.SchreckerT.Governing Global Health: Challenge, Response, InnovationAldershotAshgate200710.7748/ns2007.10.22.7.30.b68226983028

159
Price-SmithAndrewContagion and Chaos: Disease, Ecology and National Security in the Era of GlobalizationCambridge, Mass.MIT Press2009

160
LoaderIanWalkerNeilCivilizing SecurityCambridgeCambridge University Press2007

161
BeckUlrichRisk Society: Towards a New ModernityRitterM.LondonSage2000

162
MaplecroftVeriskAdvisory Serviceshttp://maplecroft.com/about/news/influenza_pandemic_risk_index.htmlSeptember 10, 2013

163
AlcabesPhilipDread: How Fear and Fantasy Have Fueled Epidemics from the Black Death to Avian FluNew YorkPublicAffairs2009223

164
ParkerAndrew M.VardavasRaffaeleMarcumChristopher S.GidengilCourtney A.Conscious Consideration of Herd Immunity in Influenza Vaccination DecisionsAmer. J. Prevent. Med45201311812110.1016/j.amepre.2013.02.016PMC369450223790997

165
BrilliantLarryThe Age of PandemicsWall Street Journal522009http://online.wsj.com/article/SB124121965740478983.htmlSeptember 9, 2013

166
CollierStephen J.LakoffAndrewThe Problem of Securing HealthBiosecurity Interventions: Global Health and Security in QuestionLakoffA.CollierS. J.New YorkColumbia University Press2008728

167
PepinJacquesThe Origins of AIDSCambridgeCambridge University Press2011

168
WasherPeterEmerging Infectious Diseases and SocietyBasingstokePalgrave Macmillan2010

169
GregerMichaelIndustrial Animal Agriculture’s Role in the Emergence and Spread of DiseaseThe Meat Crisis: Developing More Sustainable Production and ConsumptionD’SilvaJ.WebsterJ.LondonEarthscan2010161172

170
American Government ArchiveEmerging Diseases Drive Human, Animal Health Alliance7202007http://iipdigital.usembassy.gov/st/english/article/2007/07/20070720135159lcnirellep0.2875177.html#axzz3jqs1QYZX JacksonOxford Handbook of the History of Medicine561577

171 http://www.millenium-project.org/millenium/Global_Challenges/chall.-0.8.html, accessed August 27 2015; http://www.ophi.org.uk/wp-content/uploads/Multidimensional-Poverty-Index-2013-Alkire-Roche-and-Seth.pdf, accessed August 29, 2015.

172 http://www.who.int/whr/1998/media_centre/press_release/en/, accessed August 27, 2015.

173
JhaPrabhatAvoidable Global Cancer Deaths and Total Deaths from SmokingNature Rev. Cancer920096556641969309610.1038/nrc2703 EditorialCardiovascular Disease in China: Why Should We Care?J. Asian Health2012http://journalofasianhealth.com/article.phpNovember 5, 2012

174
HuFrank B.Globalization of Food Patterns and Cardiovascular Disease RiskCirculation1182008191319141898131110.1161/CIRCULATIONAHA.108.808493 ZimmetP.Globalization, Coca-colonization and the Chronic Disease Epidemic: Can the Doomsday Scenario Be Averted?J. Internal Med24720003013101076244510.1046/j.1365-2796.2000.00625.x

175
ArnoldDavidDiabetes in the Tropics: Race, Place and Class in India, 1880–1965Soc. Hist. Med222009245261

176 Hu, “Globalization of Food Patterns” (n. 174).

177
KentMary M.YinSandraControlling Infectious DiseasesPopul. Bull61200639

178
BaumanZygmuntGlobalization: The Human ConsequencesCambridgePolity1998 AppaduraiArjunFear of Small Numbers: An Essay on the Geography of AngerDurham, N.C.Duke University Press2006

179
CarmichaelAnn G.Plague and the Poor in Renaissance FlorenceCambridgeCambridge University Press1986 SlackPaulThe Impact of Plague in Tudor and Stuart EnglandOxfordClarendon1985

180
BhugraDineshMastrogianniAnastasiaGlobalisation and Mental DisordersBrit. J. Psychiatry184200410201470222210.1192/bjp.184.1.10

181
RingenSteinKwonHuck-juYiIlcheongKimTaekyoonLeeJoohaThe Korean State and Social Policy: How South Korea Lifted Itself from Poverty to Affluence and DemocracyOxfordOxford University Press2011

182
CuetoMarcosThe Origins of Primary Health Care and Selective Primary HealthcareAmer. J. Pub. Health942004186418741551422110.2105/ajph.94.11.1864PMC1448553

183
FangXiaopingBarefoot Doctors and Western Medicine in ChinaRochester, N.Y.Rochester University Press2012 SidelW.The Barefoot Doctors of the People’s Republic of ChinaNew Engl. J. Med286197212921300440195210.1056/NEJM197206152862404

184
KimJ. Y.MillenJ. V.IrwinA.GershmanJ.Dying for Growth: Global Inequality and the Health of the PoorMonroe, MaineCommon Courage Press2000

185
JenningsMichaelSurrogates of the State: NGOs Development and Ujamaa in TanzaniaBloomfield, Conn.Kumarian Press2007

186
KickbuschIlonaThe Development of International Health Policies—Accountability Intact?Soc. Sci. Med5120009799891097244010.1016/s0277-9536(00)00076-9

187
PollockAllyson M.NHS plc: The Privatisation of Our Health CareLondonVerso2004

188
PorterGreatest Benefit to Mankind717718

189
World Health OrganizationWorld Health Statistics. Part III: Global Health IndicatorsGenevaWHO2012

190
BivinsRobertaAlternative Medicine?: A HistoryOxfordOxford University Press2007173174181182 IllichIvanLimits to Medicine: Medical Nemesis: The Expropriation of Health1976LondonPenguin1988

191
NaSeonsamEast Asian Medicine in South KoreaHarvard Asia Quart1420124456 TaylorKimDivergent Interests and Cultivated Misunderstandings: The Influence of the West on Shaping Modern Chinese MedicineSoc. Hist. Med17200493112

192
BanerjeeMadhulikaPower, Knowledge, Medicine: Ayurvedic Pharmaceuticals at Home and in the WorldHyderabadOrient Longman2009 BodeMaartenTaking Traditional Knowledge to the Market: The Modern Image of the Ayurvedic and Unani Industry 1980–2000HyderabadOrient Blackswan2008

193
HsuE.StollbergG.Globalizing Chinese MedicineMed. Anthrop28200910.1080/0145974090284817019404879 JenningsMichaelChinese Medicine and Medical Pluralism in Dar es Salaam: Globalisation or Glocalisation?Internat. Relations192005457473

194
WujastykDagmarSmithFrederick M.Modern and Global Ayurveda: Pluralism and ParadigmsAlbanyState University of New York Press2008

195
PordiéLaurentTibetan Medicine in the Contemporary World: Global Politics of Medical Knowledge and PracticeLondonRoutledge2008

196 Author’s observation of Qinghai Red Cross Hospital, Xining, People’s Republic of China, December 12–16, 2013.

197
GaudetT. W.Integrative Medicine: The Evolution of a New Approach to Medicine and to Medical EducationIntegrative Med119986773

198
Neglected Korean Traditional Medicine: A Series of Shut-DownsSBS News11232012http://m.sbs.co.krJanuary 7, 2013

199
HorowitzMichael D.RosenweigJeffrey A.JonesChristopher A.Medical Tourism: Globalization of the Healthcare MarketplaceMedscape Gen. Med920073342PMC223429818311383

200
HardimanDavidMukharjiProjit BihariMedical Marginality in South Asia: Situating Subaltern TherapeuticsLondonRoutledge2012

201
ErinC. A.HarrisJ.An Ethical Market in OrgansJ. Med. Ethics2920031371381279642810.1136/jme.29.3.137PMC1733716 SavulescuJ.Is the Sale of Body Parts Wrong?J. Med. Ethics2920031381391279642910.1136/jme.29.3.138PMC1733718

202
DowardJamiePeople Smugglers Target Their Victims as Organ Donors and House SlavesObserver9262012www.observer.comOctober 22, 2012

203
TrauthMartinRich Countries Corner Supplies of Swine Flu Vaccine7262009Agence France Pressewww.afp.comOctober 22, 2012 HupstonFleurSwine Flu Vaccines Dumped in Africa9152010http://www.naturalnews.com/029742_vaccines_swine_flu.htmlOctober 22, 2012

204
de SwaanAbramIn Care of the State: Health Care, Education, and Welfare in Europe and the USA in the Modern EraNew YorkOxford University Press1988

205
IriyeAkiraGlobal Community: The Role of International Organizations in the Making of the Contemporary WorldBerkeleyUniversity of California Press2002

206
KoplanJ. P.BondT. C.MersonM. H.Towards a Common Definition of Global HealthLancet3732009199319951949356410.1016/S0140-6736(09)60332-9PMC9905260 BrownTheodore M.CuetoMarcosFeeElizabethThe World Health Organization and the Transition from ‘International’ to ‘Global’ HealthAmer. J. Pub. Health96200662721632246410.2105/AJPH.2004.050831PMC1470434

207
SkinnerQuentinThe Foundations of Modern Political Thought. Volume I: The RenaissanceCambridgeCambridge University Press197842464965 HendersonJohnPiety and Charity in Late Medieval FlorenceOxfordClarendon19941620354359 HendersonThe Renaissance Hospital: Healing the Body and Healing the SoulNew Haven, Conn.Yale University Press20062831

208
HeldDavidGlobal Covenant: The Social Democratic Alternative to the Washington ConsensusCambridgePolity2004 WaldronJ.What Is Cosmopolitan?J. Polit. Philos82000227243

209
LoaderWalkerCivilizing Security258264

